# Lipid trajectories improve risk models for Alzheimer’s disease and mild cognitive impairment

**DOI:** 10.1016/j.jlr.2024.100714

**Published:** 2024-11-23

**Authors:** Bruce A. Chase, Roberta Frigerio, Chad J. Yucus, Smita Patel, Demetrius Maraganore, Alan R. Sanders, Jubao Duan, Katerina Markopoulou

**Affiliations:** 1 Information Technology, Endeavor Health, Skokie, IL, USA; 2 Pritzker School of Medicine, Chicago, USA; 3 Research Institute, Endeavor Health, Evanston, IL, USA; 4 Department of Neurology, Endeavor Health, Evanston, IL, USA; 5 Department of Neurology, Tulane University School of Medicine, New Orleans, LA, USA; 6 Center for Psychiatric Genetics, Endeavor Health Research Institute, Evanston, IL, USA; 7 Department of Psychiatry and Behavioral Neuroscience, University of Chicago, Chicago, IL, USA; 8 Department of Neurology, Pritzker School of Medicine, University of Chicago, Chicago, IL, USA

**Keywords:** Alzheimer’s disease, cholesterol, LDL, lipids, triglycerides, HDL-C, *APOE*, polygenic risk score, group-based trajectory analysis, variability independent of the mean

## Abstract

In this retrospective, case-control study, we tested the hypothesis that blood-lipid concentrations during the decade prior to cognitive symptom onset can inform risk prediction for Alzheimer's disease (AD) and stable mild cognitive impairment (MCI). Clinically well-characterized cases were diagnosed using Diagnostic and Statistical Manual of Mental Disorders, Fourth Edition (DSM-IV) criteria; MCI cases had been stable for ≥5 years; and controls were propensity matched to cases at symptom onset (MCI: 116 cases, 435 controls; AD: 215 cases, 483 controls). Participants were grouped based on (i) longitudinal trajectories and (ii) quintile of variability independent of the mean (VIM) for total cholesterol, HDL-C, low-density lipoprotein cholesterol, non-HDL-C, and ln(triglycerides). Risk models evaluated the contributions of lipid trajectory and VIM groups relative to *APOE* genotype or polygenic risk scores (PRSs) for AD and lipid levels and major lipoprotein confounders: age, lipid-lowering medications, comorbidities, and other longitudinal correlates of blood-lipid concentrations. In models with AD-PRS, higher MCI-risk was associated with the two lower HDL-C trajectories [odds ratios: 3.8(1.3−11.3; *P* = 0.014), 3.2(1.1−9.3; *P* = 0.038), relative to the high trajectory], and the lowest VIM quintile of non-HDL-C [odds ratio: 2.2 (1.3−3.8: *P* = 0.004), relative to quintiles 2−5]. Higher AD-risk was associated with the two lower HDL-C trajectories [odds ratios: 2.8(1.5−5.1; *P* = 0.001), 3.7 (2.0−7.0; *P* < 0.001)], and the lowest VIM quintile of total cholesterol [odds ratio: 2.5(1.5−4.0: *P* < 0.001)]. Inclusion of lipid-trajectory and VIM groups improved risk-model predictive performance independent of *APOE* and AD or lipid-level PRSs, providing important real-world perspectives on how longitudinal levels and variation of blood-lipid concentrations contribute to risk of cognitive decline.

## Graphical abstract

**Figure:**
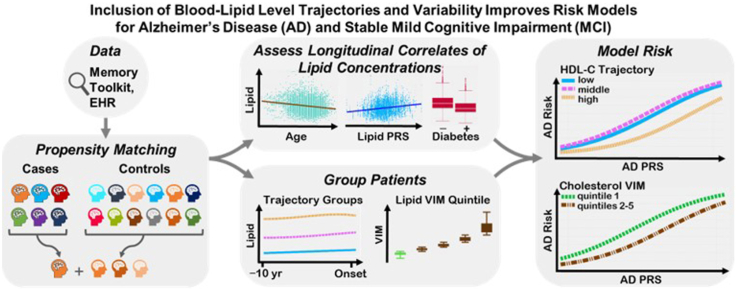

Alzheimer’s disease (AD) is the most prevalent neurodegenerative disorder, affecting almost 58 million people globally, including an estimated 6 million in the United States (1, 2). Mild cognitive impairment (MCI) typically precedes the onset of dementia, but may not progress to AD; some individuals with MCI either do not experience further cognitive decline or revert to normal cognition (3).

Mounting genetic and biological evidence suggests a central role for lipid dysregulation in AD and other neurodegenerative conditions (4, 5, 6, 7, 8, 9, 10, 11, 12, 13, 14). *APOE* ε4, the strongest genetic risk factor for both early and late onset AD, is associated with increased plasma total cholesterol (TC) (which includes LDL-C, HDL-C, and VLDL-C)—primarily due to an increase in LDL-C. Variants of *APOB*, which encodes the main lipoprotein in LDL-C, are associated with early onset AD (15), and overexpression of *ApoB* in mice results in hyperlipidemia, neurodegeneration, increased APP expression, and amyloid plaques (16, 17). The *APOE*-ε4 allele disrupts brain cholesterol homeostasis (18, 19, 20, 21) and is associated with the accumulation of lipid droplets in glia (22) and tau phosphorylation in neurons (23). Furthermore, the lipid composition of amyloid plaques differs between AD cases and cognitively unaffected individuals (24).

Many studies have reported associations between blood lipids, lipoproteins, and apolipoproteins, and dementia risk, although they are often conflicting (25). Some studies have suggested that high TC and LDL-C levels during midlife are associated with increased risk of AD (26, 27, 28), including early onset AD (15), and that high TC levels are associated with an increased cerebral burden of Aβ (29). Other studies suggest that low TC and LDL-C levels in later life are associated with an increased dementia risk (30, 31), although other studies showed no such correlation (32). Similarly, whereas higher serum triglyceride levels in older adults may reduce AD risk and slow cognitive decline, higher levels in midlife are associated with abnormal Aβ and tau levels two decades later (28, 33, 34), although these risks may be modified by *APOE* genotype (35). Other reports have suggested that increased variability in metabolic parameters (36), including lipid levels (37, 38), over time is associated with increased dementia risk.

Several risk factors for AD, including aging, education level, and socioeconomic status are also associated with changes in serum lipid levels (39), and comorbidities such as atherosclerosis and diabetes are associated with both lipid concentrations and AD- and MCI-risk. Dementia risk has also been associated with specific types of serum triglycerides (40) and with dietary fat intake (41). A high cholesterol level at midlife is associated with obesity, metabolic syndrome, and diabetes, which are all AD risk factors. In contrast, cholesterol levels at older ages may reflect loss of body mass and fat tissue, as well as use of cholesterol-lowering medicines.

The complexity of the interrelationship between blood lipid levels, lipid-associated comorbidities, metabolism, aging, and genetic factors and risk of cognitive dysfunction is clear; however, the mechanisms underlying the role of blood-lipid levels in neurodegeneration are not fully understood. Although peripheral and brain cholesterol levels may vary independently of each other due to the presence of the blood-brain barrier, which is impermeable to cholesterol, serum cholesterol levels may nonetheless affect brain cholesterol metabolism (42).

It may be clinically useful to understand the extent to which blood-lipid levels—in particular longitudinal levels before disease onset—interplay with genetic and nongenetic factors to predict AD and MCI risk. A series of cross-sectional metabolomic studies using mass spectrometry or nuclear magnetic resonance spectroscopy have identified specific blood metabolites, lipids, apolipoproteins, and lipoproteins that could serve as potential biomarkers for AD diagnosis, prognosis, patient monitoring, or as therapeutic targets (reviewed in (43) see also (7, 44, 45)). Such studies have provided insight into the role of specific molecules and lipid fractions, such as HDL-4, which is positively associated with AD pathophysiology (46), VLDL-C, and intermediate-density lipoprotein cholesterol; have demonstrated that coregulated serum lipids are associated with AD pathophysiology (47); and have examined the mechanistic relationship between AD risk conferred by *APOE* genetic status and blood lipid species. However, as these techniques are not routinely available in a clinical setting, these potential biomarkers are not currently available to clinicians for prediction or diagnosis of dementia (48, 49).

We sought to test the hypothesis that blood-lipid data obtained as part of routine clinical management prior to cognitive-symptom onset can inform risk prediction for AD or stable MCI. For this, we undertook a retrospective case-control study to examine blood levels of TC, HDL-C, non-HDL-C, LDL-C, and triglycerides, and their variation over time in clinically and genetically well-characterized patient cohorts from a real-world, community-practice setting. After propensity matching controls with cases at age of first cognitive symptom, we identified longitudinal correlates of blood-lipid concentrations and calculated two distinct measures of lipid level variation—relative levels and variability independent of the mean (VIM)—during the decade prior to symptom onset. We then modeled AD- and MCI-risk to evaluate the relative contributions of lipid trajectories and VIM, the longitudinal correlates of blood-lipid concentrations, genetic risk, patient demographics, and medical history.

## Materials and Methods

### Study overview

In this retrospective, clinical, and genetic case/control study, we assessed the contributions of serum cholesterol and lipid concentrations over the ten years before cognitive symptom onset to risk of AD or stable MCI, relative to other risk factors and patient characteristics associated with longitudinal lipid concentrations. Controls—patients without neurodegenerative disease—were propensity matched to cases at symptom onset. We used two distinct approaches to characterize blood-lipid concentrations of TC, HDL-C, LDL-C, non-HDL-C, and triglycerides in the case/control cohorts.

First, we used nonparametric group-based trajectory models to identify sets of patients sharing similar longitudinal lipid trajectories over time. Second, we calculated the VIM—a measure that is robust to heteroscedasticity and uncorrelated with mean lipid levels—for each lipid type. In unadjusted analyses, we assessed the magnitude of the association between trajectory group or lipid VIM quintile with MCI or AD risk and examined other patient characteristics associated with longitudinal lipid levels, MCI, or AD. We then used covariate-adjusted logistic regression to model the contribution of each lipid trajectory group and VIM quintile to AD or MCI risk in the context of genetic factors, patient demographics, patient medical history, and the longitudinal correlates of lipid concentration. This allowed us to infer whether the AD- and MCI-risk associated with different lipid trajectories or VIM groups retained significance after controlling for genetic factors, other variables influencing AD- and MCI-risk, and lipid concentrations.

### Study patients, diagnostic criteria, case/control matching, and data elements

We analyzed data obtained from patients enrolled in *The DodoNA Project: DNA Predictions to Improve Neurological Health* (DodoNA) (N = 12,498), which investigates the contribution of genetic risk to progression and outcomes in ten neurological conditions (50, 51, 52, 53, 54, 55, 56, 57, 58, 59, 60, 61, 62, 63) and in an at-risk *Brain Health* cohort (58) (supplemental Table S1). Each DodoNA cohort is followed longitudinally using structured clinical documentation support (SCDS) tools embedded in the electronic health record (EHR) to record patient data at the point of care. The DodoNA project was approved by the NorthShore University HealthSystem Institutional Review Board (EH10-139, April 26, 2011), and this work abides by the Declaration of Helsinki principles.

**Fig. 1:**
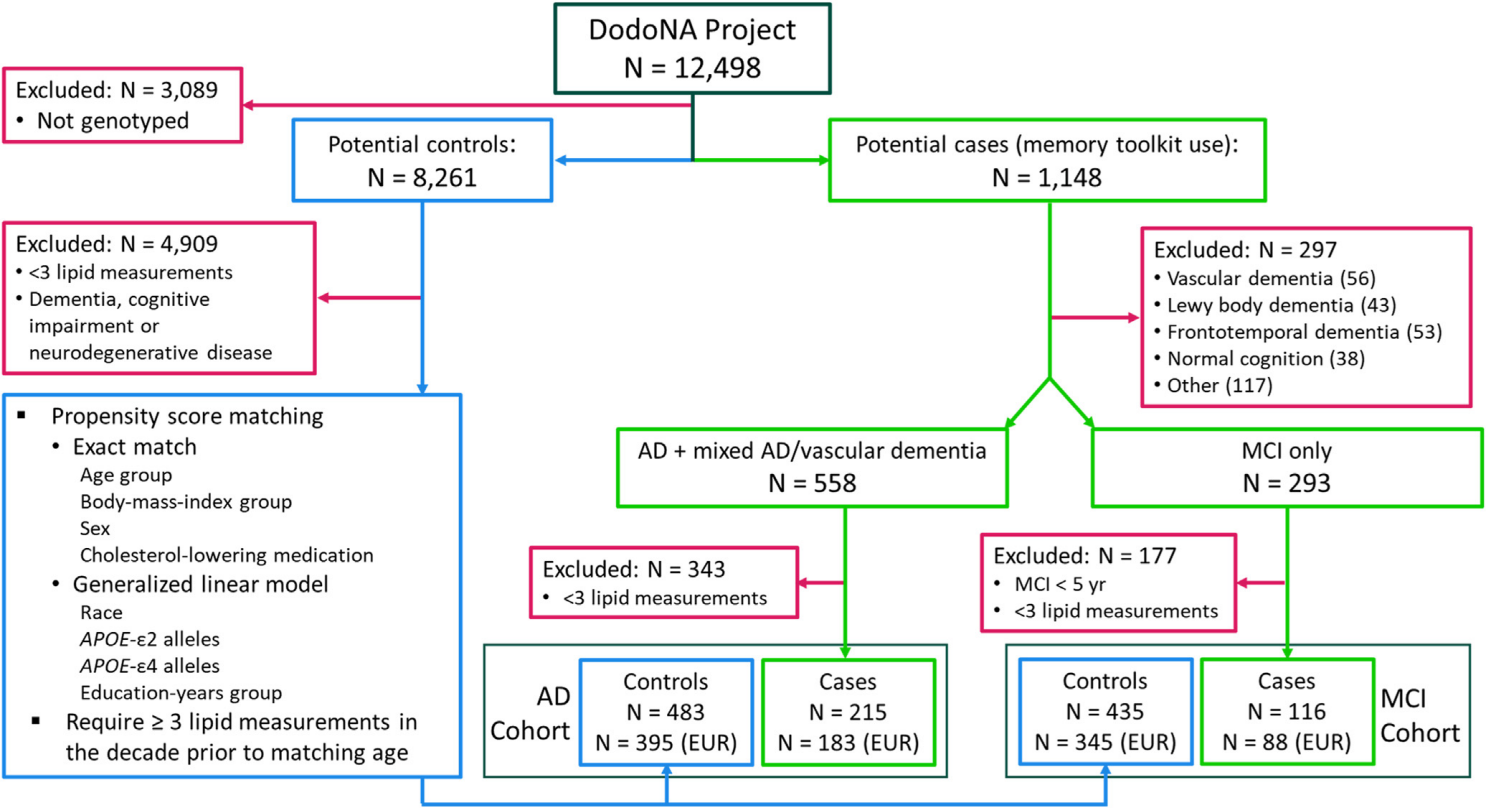
Selection of AD and MCI case/control cohorts. Case/control cohorts were selected from genotyped patients enrolled in the DodoNA project. Cases were selected from patients who were followed with a structured clinical documentation toolkit for memory disorders. AD (AD or mixed AD/vascular dementia) and MCI diagnoses utilized DSM-IV criteria. Exclusion criteria for cases were: normal cognition upon evaluation, an inconclusive diagnosis, a DSM-IV-based diagnosis of vascular dementia (only), Lewy body dementia, frontotemporal dementia, or cognitive impairment or dementia associated with another condition (parkinsonism, stroke, normal pressure hydrocephalus, cortical basal degeneration, progressive supranuclear palsy, stroke, alcohol use, end-stage-renal disease, subdural hematoma, B12 deficiency, a psychiatric disorder, or radiation therapy). AD and MCI cases were excluded if they had < 3 blood-lipid measurements in the decade prior to first symptom onset, and MCI cases were included only if MCI did not progress to dementia for at least five years. Controls were selected from patients enrolled in studies of other neurological diseases or a brain-health cohort (see supplemental Table S1). Controls did not have an ICD code for dementia or cognitive impairment (see Materials and Methods), any diagnosis of neurodegenerative disease, and had ≥3 blood lipid measurements. Cases were propensity-score matched to controls based on the criteria shown and controls were retained only if they had ≥3 blood-lipid measurements in the decade prior to their matching age. The number of cases and controls in each cohort, and the number with European ancestry (EUR), is shown. AD, Alzheimer's disease; DSM, Diagnostic and Statistical Manual of Mental Disorders; ICD, International Classification of Disease; MCI, mild cognitive impairment.

We analyzed clinical data collected from April 26, 2011 through February 24, 2024, and data on lipid measurements, International Classification of Disease (ICD) codes, and social history stored in our Electronic Data Warehouse (EDW) from its inception in 2003 through February 24, 2024. Cases with AD or MCI, selected from DodoNA patients who were diagnosed using DSM-IV criteria (64), were followed using a memory SCDS toolkit (56); the data elements collected are summarized in supplemental Table S2. Patients who had at least three annual serum lipid measurements, in different years, during the 11 years prior to, and including the year of symptom onset in cases, or age at match in controls, were included in the study. Patients with fewer than three lipid measurements were excluded (Fig. 1).

Blood-lipid concentrations were determined using standard clinical assays. LDL-C and non-HDL-C concentrations were calculated as described (65) if they were not recorded in the EDW. Triglyceride values were log-transformed to normalize their distribution. Use of blood cholesterol-lowering medications—any statin, gemfibrozil, fenofibrate, clofibrate, bempedoic acid, ezetimibe, cholestyramine, colestipol, colesevelam, alirocumab, or evolocumab—was analyzed over the same 11-year period. We inferred that a patient was using this type of medication at the time of blood lipid measurement if, before or during that time, a medication order was listed as "Sent", "Dispensed" or "Verified", but not "Discontinued", unless the order was replaced by another medication. Use of cholesterol-lowering medication was included as a time-dependent covariate in group-based trajectory models, in assessing longitudinal correlates of lipid concentrations, and as a criterion for matching cases and controls; however, lipid data were not adjusted for medication use.

We analyzed data from two cohorts (Fig. 1): (i) the AD cohort included cases [N = 215 (183 European Ancestry (EUR))] and matched controls [N = 483 (395 EUR)]. Cases had a diagnosis of AD (N = 190) or mixed AD and vascular dementia (VaD) (N = 25). Patients with only VaD or with non-AD dementia (e.g., Pick's disease, frontotemporal dementia, Lewy Body dementia, and so on) were excluded. (ii) The MCI cohort included cases [N = 116 (88 EUR)] and matched controls [N = 435 (345 EUR)]. Cases had a diagnosis of MCI with a duration of five or more years, with no diagnosis of another dementia or neurodegenerative disease. Since more than half of MCI cases progress to dementia within five years (66, 67, 68), we used the inclusion criterion of 5+ years with MCI to focus on individuals with stable MCI who were less likely to convert to dementia.

For both cohorts, controls had no neurologist diagnosis of any cognitive or neurodegenerative disorder or any ICD code for dementia, cognitive impairment or neurodegenerative disease (ICD-9: 290–, 294–, 323–, 330–, 331–, 332–, 333.4, 437–, 797–; ICD-10: F01–, F02–, F03–, F06–, G20–, G30–, G31–, I69–, R41–) in the EHR. Individuals included in the AD and MCI cohorts were enrolled from different DodoNA studies (see supplemental Table S1).

To generate case and control groups with similar covariate distributions for both cohorts, we propensity-matched cases at the age of first cognitive symptoms to controls using the *R* package *MatchIt* (69). Exact matches were used for sex, age group (50–59, 60–64, 65–72, 73–77, 78–81, 82–85, 86–98), BMI group (<18.5, 18.5–24.9, 25–29.9, 30–34.9, 35–40, >40 kg/m^2^), and use of cholesterol-lowering medication. Nearest matches, selected using a generalized linear model (logistic regression), were used for patient-reported race, number of *APOE-*ε2 and -ε4 alleles, and patient-reported years of education (<12, 12, 12–15, ≥16 years).

The following were recorded as binomial variables: whether patients were smokers or moderate-to-heavy alcohol users (>7 drinks/week) prior to the matching age, based on patient-reported social history information stored in the EDW; and the presence of an ICD9 or ICD10 code for hypertension (401– to 403–, 405–, I10– to I16–) or atherosclerosis (414–, 437–, I25–), cerebrovascular disease (437, I67.3, I69–), diabetes (249–, 250–, E09– to E11–, E13–), ischemic heart disease/myocardial infarction (21.29, 410– to 414–, 429.2, I20– to I25–, I31.2, I51.3, I51, I82.9, I97–, I99.8, Q21–, Q89.9, Z13.6), or malignant neoplasm (140– to 239–, C00–– to C96––, D00–– to D49––, H47–).

### Genotyping, imputation, and calculation of polygenic risk scores

Single nucleotide polymorphism genotyping on an Affymetrix Axiom™ array with custom content and quality control was performed as previously described (60). We used the Michigan Imputation Server (https://imputationserver.sph.umich.edu/) (70) for genotype imputation, phasing, and ancestry estimation [Haplotype Reference Consortium reference panel, Version r1.1 2016, Eagle v2.4, *r*^2^ = 0.8], and to calculate polygenic risk scores (PRS) from The Polygenic Score (PGS) Catalog (https://www.pgscatalog.org/) (71). In models for the longitudinal correlates of lipid levels and in the models for AD and MCI risk, we evaluated PRS for AD (PGS004034, PGS004092, both of which include variants in the *APOE* region) (72), triglycerides (PGS003147, PGS003152), TC (PGS003142, PGS003137), LDL-C (PGS003037, PGS003032), HDL-C (PGS002957) (73), and non-HDL-C (PGS002782) (74). The PRS were standardized (mean = 0, standard deviation = 1) within each case/control cohort to facilitate a comparison of effect sizes.

### Statistical analyses

Differences in demographic and medical history data elements, lipid trajectory groups, and VIM quintiles between cases and controls were evaluated using χ^2^ tests for categorical variables and Kruskal-Wallis tests for continuous variables. A Bonferroni adjustment was applied to tests of association between AD or MCI with lipid trajectory groups and VIM quintiles and to tests of differences in cohort characteristics across these groups. The distribution of PRS in cases and controls was visualized using a kernel-density plot, obtained using an Epanechnikov kernel function. A Kolmogorov-Smirnov test evaluated the equality of PRS distributions in cases and controls. Inter-PRS correlations were assessed using the Pearson correlation method. Statistical analyses were performed using *Stata* BE 18 or 18.5 (https://www.stata.com/) and *R* (https://www.r-project.org/).

### Longitudinal correlates of lipid levels

Prior to developing group-based trajectory models, we used multilevel mixed-effects models to determine whether our patient population showed similar longitudinal correlates of lipid concentrations as those described by Duncan *et al.* (65) and to evaluate associations between genetic factors and longitudinal lipid concentrations. By analyzing data from the same individual over time as well as across individuals, we were able to estimate the impact of covariates on temporal patterns at the study-sample level as well as the overall pattern of change over time at the individual level. These models included a random intercept and random slope for age (to account for different initial lipid levels in each participant and a different slope for age). The models used maximum likelihood estimation as a maximization criterion, with an unstructured covariance matrix that allowed for correlation between the random slopes and intercepts for age. Covariates initially evaluated were selected based on those identified in (65) for which data were available in the EHR. They included age, sex, cholesterol-lowering medication use, BMI, smoker, years of education (dummy coded as <12 years, 12 years, >12 years), moderate-to-heavy alcohol use; ICD-code based diagnoses (as described above) of hypertension, cerebrovascular disease, diabetes, ischemic heart disease or myocardial infarction, atherosclerosis, and malignant neoplasm; interactions between age and sex, BMI, diabetes, hypertension, cerebrovascular disease, or atherosclerosis; interactions between alcohol use and smoking; and interactions between sex and diabetes or lipid-lowering medication use. We also included genotypes at *APOE*-ε4 and ε2, *PICALM* AA, and *CLU* CC, the set of PRS for AD and blood lipids described above, and 10 genetic principal components as covariates. Since these analyses were aimed at informing the selection of covariates that would later be used in AD and MCI risk models, we evaluated these covariates in all patients, not only those with EUR ancestry. The best fitting model was identified using Akaike information criterion (AIC) backward selection.

### Group-based trajectory models

Group-based lipid trajectory models were developed using the semiparametric, model-based clustering method described by Jones and Nagin (75). This method allows for the inclusion of individuals with multiple measurements that contain gaps, i.e., that are not sequential, and estimates the probability of group membership for each individual. The models were produced by fitting longitudinal lipid concentration data to finite-mixture models using the *traj* plug-in in *Stata*/BE 18.0 (https://www.andrew.cmu.edu/user/bjones/traj) (76). We evaluated model fit with two to five trajectory groups for each dependent variable (lipid measurement) over time (years prior to year of first symptom in cases or age match in controls); use of cholesterol-lowering medication was included as a time-varying covariate. The link function between time and the lipid measurement variable was censored-normal (a tobit model). The best-fitting models were identified through an iterative process using the fit-criteria assessment plot developed by Klijn *et al.* (77), as described previously (78). Briefly, the “best” model was identified by fitting single- to five-group models with zero to fourth-order polynomials and comparing model fit based on Bayesian Information Criteria, AIC, and model-maximized likelihood. Following Nagin (79), we required each trajectory group to have an average posterior probability of group assignment ≥ 0.85 and odds of correct classification ≥5.0. To maintain clinical relevance, we also required that each group included at least 5% of a case/control cohort.

### Variability independent of the mean

VIM assesses variation over time that is uncorrelated to the mean and is robust to heteroscedasticity. To address whether lipid variability, over the decade prior to cognitive symptom onset, was associated with disease risk, we calculated VIM for each lipid species, as described by Moser *et al.* (38), using the same lipid measurements that were used to develop group-based trajectory models. We then tested associations between lipid VIM quintile and AD or MCI onset.

### AD and MCI risk models

Covariate-adjusted logistic regression risk models were developed using AIC backward selection, and cluster-robust standard errors that allowed for intramatching-group correlation. Models with PRS as covariates (case ∼ covariates + lipid-trajectory groups + 10 genetic principal components + PRS) only included individuals with EUR ancestry, as most PRS were developed in populations with this ancestry, while models with *APOE* genotypes as covariates (case ∼ covariates + lipid-trajectory groups + *APOE* genotypes) included entire case/control cohorts. Covariates considered in all models included age and matching weights as continuous variables, and female sex and >12 years of education as binomial variables. Other binomial variables included smoking, moderate-to-heavy alcohol use, hypertension, cerebrovascular disease, diabetes, atherosclerosis, or ischemic heart disease, prior to the year of first symptom (for cases), or age-match (for controls). Other categorical variables included trajectory group assignments and VIM quintiles for non-HDL-C, HDL-C, ln(triglycerides), LDL-C, and TC. PRS were evaluated as continuous variables.

When developing risk models, we considered previously reported correlations between some AD and lipid PRS (42) and expected correlations between PRS for certain lipids by evaluating inter-PRS correlations. In the population studied, different PRS for the same trait (AD, triglycerides, LDL, or TC) showed very strong positive correlations (*r* = 0.8−1.0) and different PRS for TC, LDL-C, and non-HDL-C showed strong to very strong positive correlations (*r* = 0.6−0.9); however, both AD PRS showed a very weak positive correlation (*r* = 0.0−0.2) with PRS for different lipids. For PRS showing strong positive correlations, we evaluated each PRS individually to identify the best model fit. For example, compared to PGS003142_TC_ and PGS004034_AD_, PGS003137_TC_ and PGS004092_AD_, respectively, gave better model fit (by AIC) and higher pseudo-R^2^, so these PRS were retained in the final models.

Models considering *APOE* genotypes evaluated *APOE-*ε4 and *APOE-*ε2, each relative to the no-*APOE-*ε4 or no-*APOE-*ε2 allele class, and race, dummy-coded as Caucasian, Black/African American, or Asian. Models also evaluated interactions of age with atherosclerosis, hypertension, cerebrovascular disease, PGS004092_AD_, and HDL-C trajectory groups. All models passed link and Hosmer-Lemeshow goodness-of-fit tests. The area under the receiver operator characteristic was calculated for risk models using *roccomp* in *Stata* BE 18.5. We report the Bonferroni-corrected *P* value of a test of equality of area under the curves produced by each set of related models: (1) baseline covariates such as age, sex, and genetic principal components; (2) baseline covariates plus lipid groups; (3) baseline covariates plus genetic factors (PRS or *APOE* genotypes); and (4) baseline covariates, lipid groups, and genetic factors.

## Results

### Patient demographics and lipid measurements

Similarities and differences in the demographics, *APOE* allele status, and medical history for the matched cases and controls are summarized in Table 1; genetic ancestry is summarized in supplemental Table S3. Our study population was more than 91% Caucasian and approximately 50% female. Propensity-matched cases and controls were similar except for three characteristics: i) As expected, *APOE*-ε4 homozygotes and heterozygotes were more frequent in both AD and MCI cases than in controls; ii) although over 60% of controls and cases in both cohorts had at least 16 years of education, the AD cohort had more cases with 12 years of education than controls; iii) the AD cohort had more cases with history of cerebrovascular disease than controls, and the MCI cohort had fewer cases with a history of atherosclerosis than controls.

**Table 1:** Demographics in AD and MCI case/control cohorts

Characteristic, *type of match*^a^	AD Cohort			MCI Cohort		
	Cases	Controls	*P*^b^	Cases	Controls	*P*^b^
	N = 215	N = 483		N = 116	N = 435	
Sex, *exact*						
Male	103 (47.9)	240 (49.7)	0.664	58 (50)	233 (53.6)	0.495
Female	112 (52.1)	243 (50.3)		58 (50)	202 (46.4)	
Age group, *exact*^c^						
50–59	7 (3.3)	19 (3.9)	0.154	3 (2.6)	20 (4.6)	0.873
60–64	3 (1.4)	10 (2.1)		1 (0.9)	3 (0.7)	
65–72	36 (16.7)	116 (24.0)		28 (24.1)	108 (24.8)	
73–77	42 (19.5)	97 (20.1)		28 (24.1)	113 (26.0)	
78–81	48 (22.3)	112 (23.2)		23 (19.8)	83 (19.1)	
82–85	44 (20.5)	69 (14.3)		21 (18.1)	59 (13.6)	
86–98	35 (16.3)	60 (12.4)		12 (10.3)	49 (11.3	
BMI group, *exact*						
<18.5	3 (1.4)	11 (2.3)	0.943	3 (2.6)	8 (1.8)	0.878
18.5–24.9	91 (42.3)	195 (40.4)		37 (31.9)	152 (34.9)	
25–29.9	90 (41.9)	205 (42.4)		48 (41.4)	186 (42.8)	
30–34.9	27 (12.6)	62 (12.8)		17 (14.7)	60 (13.8)	
35–40	4 (1.9)	10 (2.1)		9 (7.8)	22 (5.1)	
>40	0 (0)	0 (0)		2 (1.7)	7 (1.6)	
Blood-cholesterol lowering medication use, *exact*						
Used^b^	158 (73.5)	365 (75.6)	0.558	89 (76.7)	348 (80.0)	0.439
Race, *nearest*						
Caucasian	203 (94.4)	442 (91.5)	0.592	107 (92.2)	397 (91.3)	0.814
Black/African-American	5 (2.3)	15 (3.1)		2 (1.7)	10 (2.3)	
Asian	6 (2.8)	23 (4.8)		5 (4.3)	24 (5.5)	
Other	1 (0.5)	3 (0.6)		2 (1.7)	4 (0.9)	
*APOE* ε2 alleles, *nearest*						
0	198 (92.1)	419 (86.8)	0.082	100 (86.2)	381 (87.6)	0.586
1	17 (7.9)	60 (12.4)		15 (12.9)	53 (12.2)	
2	0 (0)	4 (0.8)		1 (0.9)	1 (0.2)	
*APOE* ε4 alleles, *nearest*						
0	102 (47.4)	327 (67.7)	<**0.001**	73 (62.9)	316 (72.6)	**0.017**
1	86 (40)	139 (28.8)		36 (31.0)	111 (25.5)	
2	27 (12.6)	17 (3.5)		7 (6.0)	8 (1.8)	
Years of education, *nearest*						
<12	5 (2.3)	14 (2.9)	**0.026**	3 (2.6)	9 (2.1)	0.387
12	45 (20.9)	58 (12.0)		22 (19.0)	56 (12.9)	
12–15	32 (14.9)	81 (16.8)		20 (17.2)	85 (19.5)	
≥16	133 (61.9)	330 (68.3)		71 (61.2)	285 (65.5)	
Smoking	1 (0.5)	11 (2.3)	0.089	3 (2.6)	9 (2.1)	0.735
Moderate-to-heavy alcohol use	21 (9.8)	52 (10.8)	0.691	13 (11.2)	50 (11.5)	0.931
Hypertension	156 (72.6)	364 (75.4)	0.433	93 (80.2)	334 (76.8)	0.437
Atherosclerosis	10 (4.7)	35 (7.2)	0.197	2 (1.7)	35 (8)	**0.016**
Cerebrovascular disease	56 (26.0)	83 (17.2)	**0.007**	23 (19.8)	71 (16.3)	0.372
Diabetes	57 (26.5)	123 (25.5)	0.771	36 (31.0)	126 (29.0)	0.664
Ischemic heart disease/myocardial infarction	61 (28.4)	162 (33.5)	0.176	38 (32.8)	165 (37.9)	0.305
Malignant neoplasm	75 (34.9)	205 (42.4)	0.060	45 (38.8)	188 (43.2)	0.391

*P* < 0.05 in bold.

a Cases were propensity matched to controls using a generalized linear model (logistic regression) with exact or nearest-neighbor matching performed using the *MatchIt* package in *R*. The type of match is indicated in italics beside variables used to match cases and controls.

b χ^2^ test.

c At age of symptom onset in cases, or at age at match in controls.

Data from a total of 698 (215 cases) and 551 (116 cases) individuals in the AD and MCI case/control cohorts, respectively, were available and included in the analysis. AD and MCI cases had a mean (standard deviation, range) follow-up of 6.5 (2.7, 0.2–14) and 7.8 (1.8, 5–13) years, respectively. As shown in supplemental Fig. S1, more than 95% of AD and MCI cases had first symptom onset after age 65. Among AD cases, 11.6% were diagnosed with mixed AD and VaD and 58.6% had a history of MCI.

Since this was a retrospective study, we were restricted to using blood-lipid data available in the EDW. As lipid measurements were not obtained every year for every patient, the number of available lipid measurements varied across patients. AD cases and controls had a median (range) of 5 (3–11) and 7 (3–11) lipid measurements, respectively, with a mean (range) of 63% (41%–75%) of patients having lipid measurements each year of the study. MCI cases and controls had 6 (3–11) and 7 (3–11) lipid measurements, respectively, and a mean (range) of 61% (44%–72%) of these patients had lipid measurements each year of the study (supplemental Fig. S2).

### Longitudinal correlates of lipid levels

To inform the choice of covariates used in AD and MCI risk models, we assessed longitudinal correlates of lipid levels in the AD and MCI case/control cohorts. For this, we used multilevel mixed-effects models to analyze lipid measurements from the same individual over time as well as across individuals. In these models, we evaluated similar longitudinal correlates of lipid concentrations to those previously identified (65), genotypes at *APOE*, *PICALM*, and *CLU*, and PRS for AD and lipid subtypes.

Table 2 summarizes the associations identified in the best fitting models. In the AD cohort, increasing age was positively associated with HDL-C but negatively associated with TC, LDL-C, ln(triglycerides), and non-HDL-C. Similar patterns were seen in the MCI cohort for LDL-C and non-HDL-C and, with a smaller effect size, for HDL-C. Except for ln(triglycerides) in the MCI cohort, female sex, or an interaction between female sex and age, was positively associated with each lipid subtype. Cholesterol-lowering medication use was associated with lower TC and LDL-C in both cohorts, and with non-HDL-C in the MCI cohort. In the AD cohort, an interaction between age and BMI was negatively associated with HDL-C, and positively associated with ln(triglycerides), while in the MCI cohort, BMI was negatively associated with TC, HDL-C, LDL-C, and positively associated with ln(triglycerides). In the AD cohort, smoking was negatively associated with TC and LDL-C. In both cohorts, moderate-to-heavy alcohol use was positively associated with HDL-C. Having at least 12 years of education was negatively associated with TC, LDL-C, and ln(triglycerides) in the AD cohort, and with non-HDL-C in the MCI cohort, while <12 years of education was negatively associated with HDL-C in the MCI cohort. In the AD cohort, when associations between lipids and comorbidities related to cardiovascular health (hypertension, cerebrovascular disease, diabetes, and ischemic heart disease/myocardial infarction) were significant, they showed negative associations. This general pattern was also seen in the MCI cohort for diabetes, ischemic heart disease, and atherosclerosis, although the best fitting models included interactions with age. In the MCI cohort, hypertension was positively associated with TC, LDL-C, ln(triglycerides), and non-HDL-C, but an interaction between hypertension and age showed negative associations with TC, LDL-C, and non-HDL-C. Prior work has suggested that some negative associations reflect treatment effects. Many, but not all, of these results are similar to those reported by Duncan *et al.* (65). Differences likely reflect reduced sensitivity due to our smaller sample size, the limited 11-year interval over which we assessed lipid levels, and our older patient population.

**Table 2:** Longitudinal correlates of lipid concentrations^a^

A. Alzheimer's Disease Case/Control Cohort, N = 698 (4,551 Observations over 11 years)^b^										
	Total Cholesterol		HDL-C		Non-HDL-C		LDL-C		ln (Triglycerides)	
Covariate	β [95% CI]	*P*	β [95% CI]	*P*	β [95% CI]	*P*	β [95% CI]	*P*	β [95% CI]	*P*
Age, yr	−0.82 [−1.07, −0.56]	<0.001	0.88 [0.75, 1.00]	<0.001	−1.03 [−1.28, −0.78]	<0.001	−0.90 [−1.12, −0.68]	<0.001	−0.012 [−0.015, −0.008]	<0.001
Female	−	−	14.0 [11.4, 16.6]	<0.001	8.16 [2.10, 14.2]	0.008	7.16 [1.57, 12.7]	0.012	−	−
Cholesterol-lowering medication use	−18.6 [−21.9, −15.3]	<0.001	−	−	−	−	−22.5 [−26.8, −18.3]	<0.001	−	−
Smoker	−23.0 [−40.3, −7.14]	0.005	−	−	−	−	−16.7 [−31.3, −2.05]	0.025	−	−
12 years education	−23.0 [−37.4, −8.64]	0.002	−	−	−	−	−17.1 [−29.7, −4.51]	0.008	−0.23 [−0.42, −0.043]	0.016
> 12 years education	−21.0 [−34.4, −7.58]	0.002	−	−	−	−	−18.3 [−30.1, −6.51]	0.002	−0.25 [−0.43, −0.080]	0.004
Moderate-to-heavy alcohol use	−	−	5.13 [1.73, 8.52]	0.003	−	−	−	−	−	−
Cerebrovascular disease	−8.17 [−13.9, −2.88]	0.002	−	−	−	−	−5.03 [−9.61, −0.45]	0.031	−0.10 [−0.17, −0.033]	0.004
Diabetes	−8.70 [−13.9, −3.54]	0.001	−5.53 [−8.07, −2.99]	<0.001	−	−	−6.63 [−11.1, −2.14]	0.004	0.16 [0.10, 0.23]	<0.001
Ischemic heart disease or myocardial infarction	−10.8 [−15.7, −5.95]	<0.001	−3.44 [−5.78, −1.09]	0.004	−8.61 [−13.1, −4.09]	<0.001	−7.05 [−11.3, −2.81]	0.001	−	−
Interactions										
Female x age	0.35 [0.29, 0.41]	<0.001	−	−	−	−	−	−	0.002 [0.001, 0.002]	<0.001
Male, cholesterol-lowering medication use	−	−	−0.21 [−1.72, 1.29]	0.780	−21.0 [−25.6, −16.3]	<0.001			−	−
Female, cholesterol-lowering medication use	−	−	−1.97 [−3.34, −0.60]	0.005	−14.0 [−18.2, −9.8]	<0.001	6.53 [0.91, 12.2]	0.023	−	−
BMI x age	−	−	−0.012 [−0.016, −0.0087]	<0.001	−	−	−	−	0.0004 [0.0003, 0.0005]	<0.001
Hypertension x age	−	−	−0.055 [−0.089, −0.020]	0.002	−	−	−	−	0.001 [0.0006, 0.002]	0.003
Genotypes										
*APOE-*ε2 heterozygote	−	−	0.95 [−2.35, 4.24]	0.573	−	−	−	−	−	−
*APOE-*ε2 homozygote	−	−	17.2 [4.08, 30.4]	0.010	−	−	−	−	−	−
Polygenic risk scores										
PGS003137 (total cholesterol)	23.3 [17.0, 29.6]	<0.001	−	−	−	−	−	−	−	−
PGS002957 (HDL-C)	8.84 [3.63, 14.1]	0.001	5.14 [4.07, 6.22]	<0.001	−	−	−	−	−	−
PGS002782 (Non-HDL-C)	−	−	−	−	3.91 [1.09, 6.74]	0.007	−	−	0.045 [0.015, 0.075]	0.003
PGS003037 (LDL-C)	−	−	−	−	4.97 [2.16, 7.77]	0.001	−	−	−	−
PGS003032 (LDL-C)	−	−	−	−	−	−	20.4 [14.9, 26.0]	<0.001	−	−
PGS003152 (triglycerides)	−	−	−	−	−	−	−	−	0.11 [0.082, 0.14]	<0.001

B. Mild cognitive impairment case/control cohort, N = 551 (3,653 Observations over 11 years)^c^										
	Total Cholesterol		HDL-C		Non-HDL-C		LDL-C		ln (Triglycerides)	
Covariate	β [95% CI]	*P*	β [95% CI]	*P*	β [95% CI]	*P*	β [95% CI]	*P*	β [95% CI]	*P*
Age, yr	−	−	0.48 [0.38, 0.58]	<0.001	−0.91 [−1.56, −0.26]	0.006	−0.84 [−1.44, −0.25]	0.006	−	−
Female	26.0 [20.4, 31.7]	<0.001	12.1 [9.66, 14.6]	<0.001	10.78 [5.68, 15.9]	<0.001	13.25 [7.60, 18.9]	<0.001	−	−
Cholesterol-lowering medication use	−18.0 [−22.4, −13.8]	<0.001	−	−	−17.0 [−20.9, −13.1]	<0.001	−16.1 [−19.7, −12.4]	<0.001	−	−
BMI	−1.20 [−1.80, −0.61]	<0.001	−0.66 [−0.92, −0.40]	<0.001	−	−	−0.58 [−1.09, −0.08]	<0.001	0.018 [0.011, 0.025]	<0.001
<12 years education	−	−	−9.34 [−18.0, −0.66]	0.035	−	−	−	−	−	−
12 years education	−	−	−	−	−29.1 [−46.0, −12,1]	0.001	−	−	−	−
>12 years education	−	−	−	−	−29.3 [−45.3, −13.3]	<0.001	−	−	−	−
Moderate-to-heavy alcohol use	−	−	6.59 [2.84, 10.3]	0.001	−	−	−	−	−	−
Hypertension	62.0 [3.76, 120.2]	0.037	−4.80 [−7.57, −2.31]	0.003	58.6 [5.33, 111.8]	0.031	54.4 [5.46, 103.3]	0.029	0.091 [0.008, 0.17]	0.032
Diabetes	−	−	−5.53 [−8.07, −2.99]	<0.001	−8.30 [−13.9, −2.69]	0.004	−	−	0.20 [0.13, 0.27]	<0.001
Ischemic heart disease or myocardial infarction	−13.0 [−19.1, −6.92]	<0.001	−4.94 [−7.58, −2.31]	<0.001	−8.44 [−13.8, −3.06]	0.002	−8.00 [−13.1, −2.89]	0.002	−	−
Atherosclerosis	−14.5 [-25.7, −3.3]	0.011	−	−	−	−	−	−	−	−
Interactions										
Hypertension x age	−1.40 [-1.78, −1.02]	<0.001	−	−	−0.77 [−1.41, −0.038]	0.039	−0.73 [−1.40, −0.052]	0.035	−	−
Male, diabetes	−	−	−	−	−	−	−11.4 [−18.1, −4.73]	0.001	−	−
Female, diabetes	−	−	−	−	−	−	−17.5 [−26.2, −8.86]	<0.001	−	−
Diabetes x age	−	−	−0.08 [−0.12, −0.04]	<0.001	−	−	−	−	−	−
Genotypes										
*APOE-*ε2 heterozygote	−	−	−	−	−	−	−	−	−0.012 [−0.010, −0.081]	0.805
*APOE-*ε2 homozygote	−	−	−	−	−	−	−	−	0.60 [0.10, 1.10]	0.019
Polygenic risk scores										
PGS003137 (total cholesterol)	8.97 [6.16, 11.8]	<0.001	−	−	−	−	−	−	−	−
PGS002957 (HDL-C)	−	−	5.18 [3.93, 6.43]	<0.001	−	−	−	−	−	−
PGS002782 (Non-HDL-C)	−	−	−	−	4.81 [1.51, 8.11]	0.004	−	−	−	−
PGS003037 (LDL-C)	−	−	−	−	4.62 [1.37, 7.87]	0.005	6.81 [4.43, 9.19]	<0.001	−	−
PGS003152 (triglycerides)	−	−	−	−	−	−	−	−	0.13 [0.10, 0.17]	<0.001

a Multilevel mixed-effects models analyzing blood lipid-measurement data from the same individual over time as well as across individuals were used to estimate the impact of covariates on temporal patterns at the study-sample level and the overall pattern of change over time at the individual level. Initial-model covariates evaluated included those used in (53), genotypes at *APOE*-ε4 and ε2, *PICALM* AA, and *CLU* CC, and a set of polygenic risk scores for Alzheimer's disease and blood lipids (see text), Polygenic risk scores were standardized within each case/control cohort to facilitate comparison of their effect sizes. Ten genetic principal components were included as covariates in all models. The best fitting models, identified using backwards selection and the Aikake information criterion, are presented here. All models passed a link test and a log-ratio test indicated that all models were significantly different from linear models at *P* < 0.0001.

b Covariates not retained in any model: BMI, *APOE*-ε4, <12 years of education, hypertension, diabetes x sex, diabetes x age, atherosclerosis, malignant neoplasm, smoking x moderate-to-heavy alcohol use, PGS004034 (AD), PGS004092 (AD), PGS003147 (triglycerides), and PGS003142 (total cholesterol).

C Covariates not retained in any model: cholesterol-lowering medication use x sex, age x BMI, *APOE*-ε4, smoking, cerebrovascular disease, malignant neoplasm, smoking x moderate-to-heavy alcohol use, PGS004034 (AD), PGS004092 (AD), PGS003147 (triglycerides), PGS003142 (total cholesterol), and PGS003032 (LDL-C).

When genetic associations were evaluated*, APOE*-ε4, *PICALM*, and *CLU* genotypes and PRS for AD failed to show an association with the concentration of any lipid in either cohort. In the AD cohort, *APOE*-ε2 homozygous status was positively associated with HDL-C, and in the MCI cohort, it was positively associated with ln(triglycerides), but with relatively wide 95% confidence intervals (CIs) that reflect the small number of individuals with this genotype. The most striking associations were those with lipid PRS. In both the AD and MCI cohorts, a PRS for each type of lipid showed robust positive associations with longitudinal concentrations of that lipid.

### Associations between lipid trajectories and AD and MCI risk

**Fig. 2:**
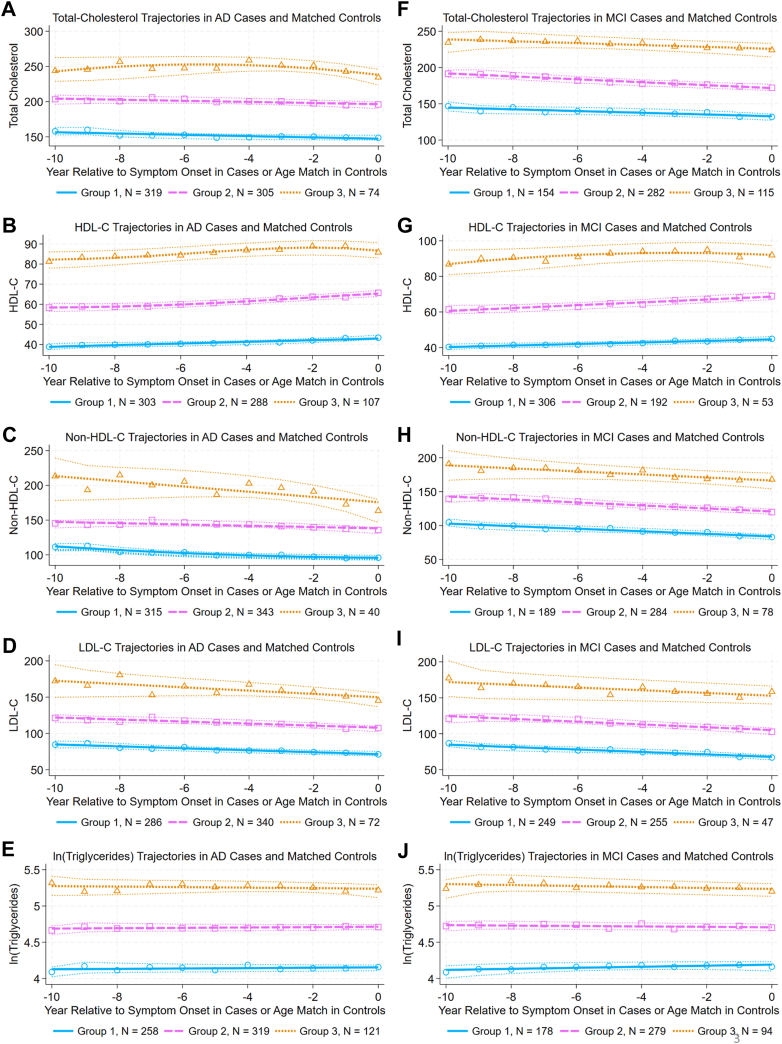
Lipid trajectories in AD and MCI case/control cohorts. Controls were propensity matched (see text and Table 2 for details) with cases at age of first symptom onset. Total cholesterol and lipid subtype measurements collected over the decade prior to the year of first symptom onset (cases) and matching age (controls) were fit to group-based trajectory models that were developed using blood-cholesterol lowering medication use as a time-dependent covariate. The plots show the best-fitting trajectory for each group for mean lipid values at each year (group 1: solid lines with open circles; group 2: long-dashed lines with open squares; and group 3: short-dashed lines with open triangles). For each trajectory, 95% confidence intervals (polynomial-fit) are indicated (dotted lines). Trajectories are shown for total cholesterol (A and F); HDL-C (B and G); non-HDL-C (C and H); LDL-C, (D and I); and ln(triglycerides), (E and J) for the AD, MCI case/control cohorts, respectively. Compare to supplemental Table S4. AD, Alzheimer's disease; MCI, mild cognitive impairment.

In both the AD and MCI case/control patient cohorts, good fits for all lipid subtypes were achieved with three trajectory-group models (Fig. 2 and supplemental Table S4). Although four-group models showed a better fit for some lipid subtypes, some groups in those models had fewer than 5% of patients, so three trajectory-group models were used in all analyses to ensure clinical relevance. In all models, trajectory group 3, corresponding to the highest levels of lipid subtype, also had the smallest number of patients and the widest CIs (Fig. 2).

The number of AD cases differed across HDL-C trajectory groups (χ^2^(2) = 13.14, *P* = 0.001, significant following Bonferroni adjustment), but not across trajectory groups for TC, non-HDL-C, LDL-C, or ln(triglycerides) (supplemental Table S5). HDL-C trajectory group 3, with the highest HDL-C levels, had relatively fewer cases (8.4%) than controls (18.4%), suggesting that the HDL-C group 3 trajectory is associated with a protective effect from AD. HDL-C trajectory group 2, with intermediate HDL-C levels, had relatively more cases (47.9%) than controls (38.3%). HDL-C trajectory group 1, with the lowest HDL-C levels, had similar numbers of cases (43.7%) and controls (43.3%). No significant difference in numbers of MCI cases was observed across trajectory groups for any lipid subtype (supplemental Table S5).

### Associations between lipid VIM quintiles and AD or MCI risk

The number of AD cases differed across VIM quintiles for TC (χ^2^(4) = 17.29, *P* = 0.002, significant after Bonferroni adjustment) and non-HDL-C (χ^2^(4) = 11.34, *P* = 0.023), with the greatest number of cases found in the lowest quintile (least variability) for each of these lipid types. The number of AD cases did not differ across VIM quintiles for HDL-C, LDL-C, or ln(triglycerides). For MCI, the greatest number of cases was found in the lowest VIM quintile for TC (χ^2^(4) = 10.92, *P* = 0.027), non-HDL-C (χ^2^(4) = 13.73, *P* = 0.008), and LDL-C (χ^2^(4) = 10.48, *P* = 0.033). The number of MCI cases did not differ across VIM quintiles for HDL-C or ln (triglycerides) (supplemental Tables S6–S10). These results suggest that lower TC and non-HDL-C variability are associated with increased risk of AD, and that lower TC, non-HDL-C and LDL-C variability are associated with increased risk of MCI.

**Fig. 3:**
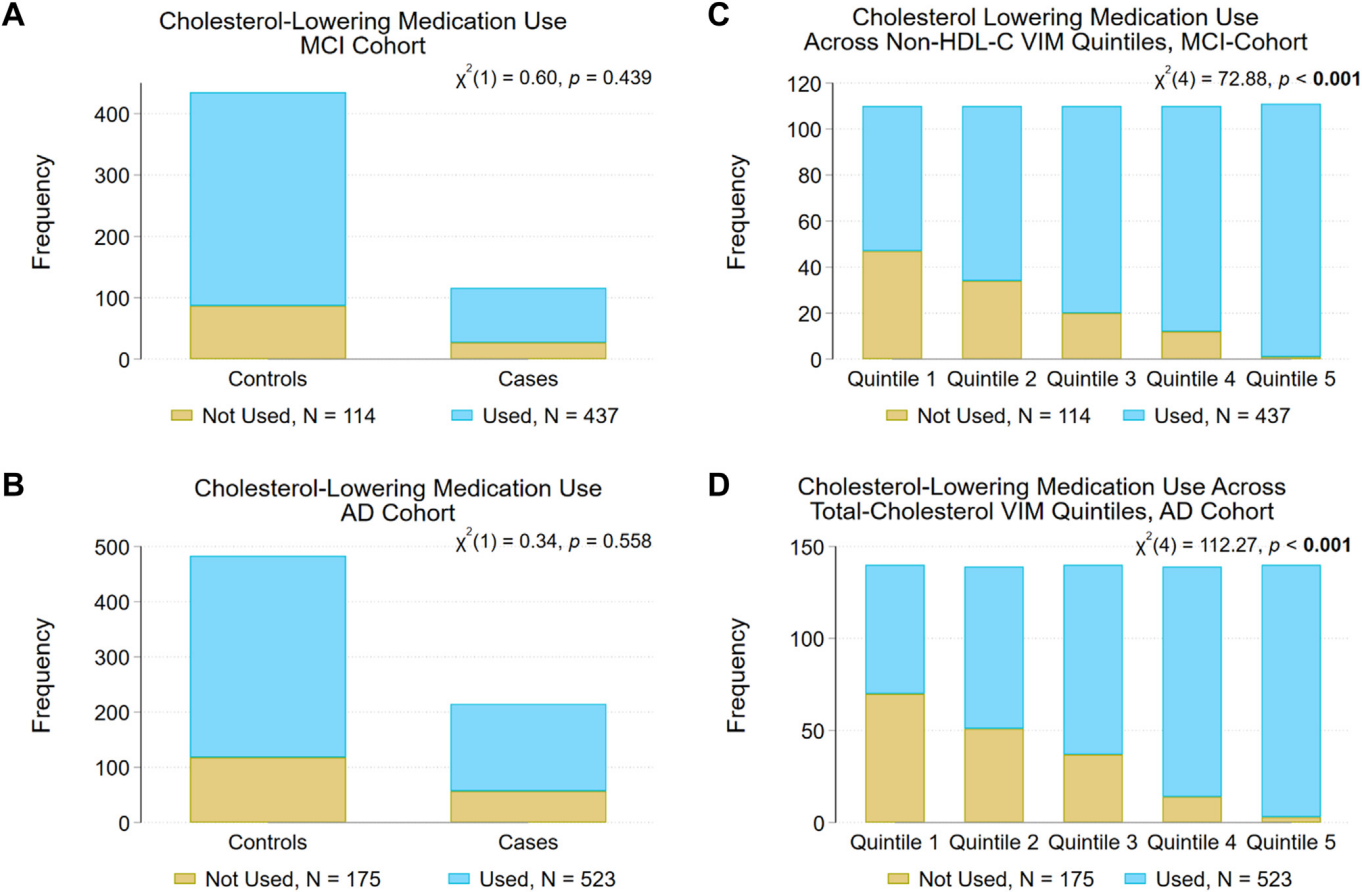
Blood-cholesterol lowering medication use in the AD and MCI case/control cohorts. Blood-cholesterol lowering medication use at age of first symptom onset (cases) did not differ from controls, at age match, in either the MCI (A) or AD (B) cohorts. Blood-cholesterol lowering medication use increased with increasing quintile of non-HDL-C variability independent of the mean (VIM) in the MCI case/control cohort (C) and with increasing quintile of total cholesterol VIM in the AD case/control cohort (D). Compare to supplemental Tables S6 and S7. AD, Alzheimer's disease; MCI, mild cognitive impairment. *P* < 0.05 in bold.

Consistent with Moser *et al.* (38), patient characteristics previously reported to be associated with longitudinal lipid levels (65) or risk of AD or MCI also differed across lipid VIM quintiles (supplemental Tables S6–S10). The characteristic that showed the strongest association with VIM quintile, and which remained significant after Bonferroni adjustment for multiple testing in each cohort, was use of cholesterol-lowering medication, which did not differ between cases and controls (Fig. 3 and Table 1). Cholesterol-lowering medication use increased in both the MCI and AD case/control cohorts with increasing VIM quintiles for TC (MCI: χ^2^(4) = 75.09, *P* < 0.001; AD: χ^2^(4) = 112.26, *P* < 0.001), non-HDL-C (MCI: χ^2^(4) = 72.88, *P* < 0.001; AD: χ^2^(4) = 113.10, *P* < 0.001) and LDL-C (MCI: χ^2^(4) = 69.11, *P* < 0.001; AD: χ^2^(4) = 99.37, *P* < 0.001).

Several other characteristics showed associations that survived Bonferroni adjustment. In the MCI cohort, an association of race with LDL-C VIM quintile (χ^2^(12) = 32.70, *P* < 0.001) reflected the near absence of non-Caucasians in the lowest quintile (supplemental Table S9). The incidence of hypertension also increased with increasing VIM quintile of TC (χ^2^(4) = 16.73, *P* = 0.002), non-HDL-C (χ^2^(4) = 24.76, *P* < 0.001), and LDL-C (χ^2^(4) = 19.13, *P* = 0.001) (supplemental Tables S6, S7, and S9). In the AD cohort, the number of patients with diabetes increased with increasing VIM quintile for TC (χ^2^(4) = 18.24, *P* = 0.001), non-HDL-C (χ^2^(4) = 24.13, *P* < 0.001) and LDL-C (χ^2^(4) = 24.82, *P* < 0.001), and the number of patients with ischemic heart disease or myocardial infarction increased with increasing VIM quintile for TC (χ^2^(4) = 33.42, *P* < 0.001) (supplemental Tables S6, S7, and S9). Although similar patterns were seen in the MCI cohort, these were not significant after Bonferroni adjustment. Associations of VIM quintile with TC, HDL-C, non-HDL-C, and LDL-C trajectory groups, *APOE*-ε4 genotype, history of malignant neoplasm, atherosclerosis, age, or alcohol use also did not retain significance after Bonferroni adjustment in the MCI cohort.

### Models for AD and MCI risk

**Fig. 4:**
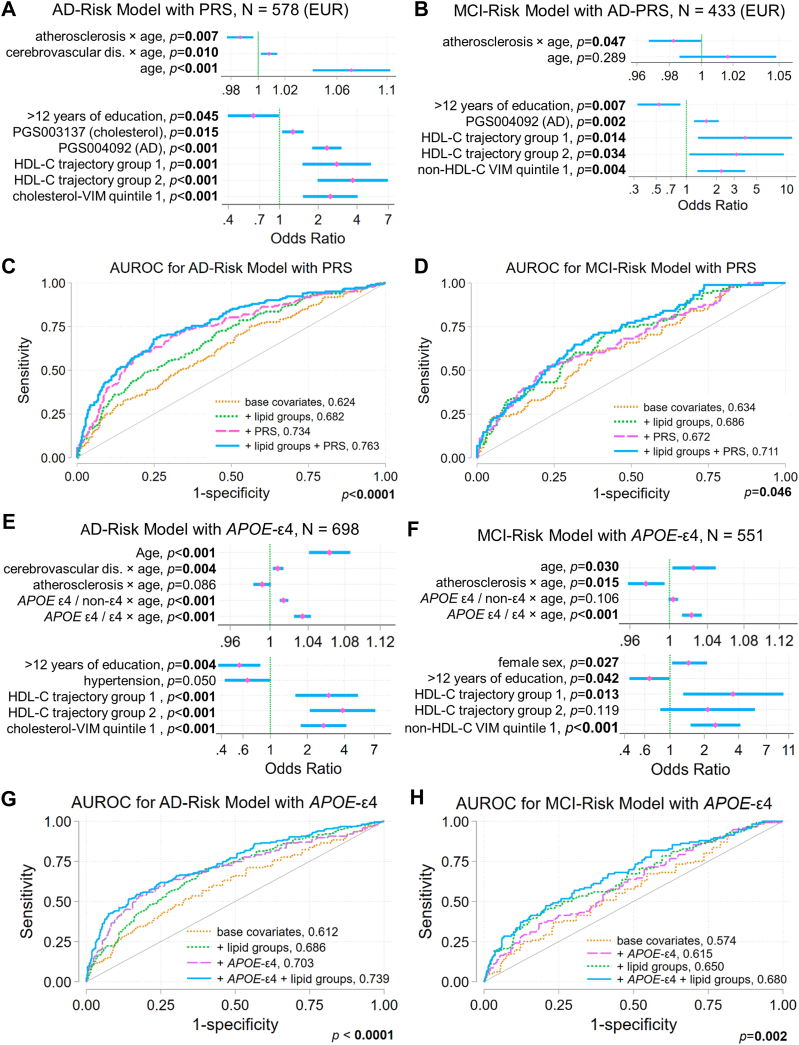
Models evaluating the contributions of lipid trajectory group and lipid variability independent of the mean (VIM) to risk of AD or MCI. Covariate-adjusted logistic regression models were developed using backward selection, and the Akaike information criterion to evaluate whether lipid trajectory groups and lipid VIM showed associations with AD or MCI risk. The covariates evaluated in these models are described in the text. Odds ratios (pink diamonds), 95% confidence intervals (blue bars), and significance are shown for covariates retained in the best fitting models for AD risk (A) and MCI risk (B) that considered polygenic risk scores for AD, including the APOE region, and lipids. Since these risk scores were mostly developed using data from genome-wide association studies in European (EUR) populations, these models were developed for the subset of study subjects with EUR ancestry. Panels E and F show odds ratios and 95% confidence intervals for covariates retained in models of AD risk (E) and MCI risk (F) that considered *APOE*-ε4 genetic status without polygenic risk scores and included all ancestries. Panels C, D, G, and H show the area under the receiver operating characteristic curve (AUROC) and scores for models with i) only the base covariates, ii) base covariates and polygenic risk scores or *APOE*-ε4 genotype, iii) base covariates with lipid groups (lipid trajectory and VIM quintile groups), and iv) base covariates, polygenic risk scores or *APOE*-ε4 genotype, and lipid groups. The Bonferroni-corrected *P* value is from a test of equality of area under these four curves. Compare panels A, B, E, and F to Table 3. AD, Alzheimer's disease; MCI, mild cognitive impairment.

We used the results from our analyses of the associations of AD and MCI with longitudinal correlates of lipid concentrations, lipid trajectory, and VIM groups to fit two covariate-adjusted risk models, one for AD and one for MCI. Table 3 and Fig. 4 show the results for the best fitting models.

**Table 3:** Modeling the contribution of lipid trajectory groups and lipid VIM to AD-risk and MCI-risk^a^

	Odds Ratio, 95% Confidence Interval	*P*
AD-risk model including polygenic risk scores		
Age	1.071, 1.041–1.103	**<0.001**
Cerebrovascular disease × age	1.008, 1.002–1.014	**0.010**
Atherosclerosis × age	0.987, 0.977–0.996	**0.007**
PGS004092 (AD)	2.333, 1.793–3.035	**<0.001**
PGS003137 (total cholesterol)	1.270, 1.048–1.539	**0.015**
>12 years of education	0.627, 0.397–0.989	**0.045**
HDL-C Trajectory Group (relative to group 3)		
1	2.781, 1.506–5.138	**0.001**
2	3.706, 1.974–6.954	**<0.001**
Cholesterol VIM quintile 1 (relative to quintiles 2–5)	2.480, 1.520–4.047	**<0.001**
AD-risk model including *APOE* genotypes		
Age	1.063, 1.041–1.086	**<0.001**
Cerebrovascular disease × age	1.008, 1.003–1.014	**0.004**
Atherosclerosis × age	0.992, 0.983–1.001	0.086
*APOE* ε4 genotype × age (relative to non-ε4 genotypes)		
*APOE* ε4 heterozygote	1.014, 1.010–1.019	**<0.001**
*APOE* ε4 homozygote	1.034, 1.025–1.043	**<0.001**
>12 years of education	0.560, 0.377–0.831	**0.004**
Hypertension	0.653, 0.426–0.999	0.050
HDL-C trajectory group (relative to group 3)		
1	2.983, 1.599–5.162	**<0.001**
2	3.868, 2.097–7.133	**<0.001**
Cholesterol VIM quintile 1	2.704, 1.765–4.144	**<0.001**
MCI-risk model including polygenic risk scores		
PGS004092 (AD)	1.580, 1.189–2.100	**0.002**
Age	1.017, 0.986, 1.048	0.289
Atherosclerosis × age	0.983, 0.967–0.999	**0.047**
>12 years of education	0.532, 0.325–0.873	**0.012**
HDL-C trajectory group (relative to group 3)		
1	3.841, 1.308–s11.28	**0.014**
2	3.159, 1.068–9.342	**0.038**
Non-HDL-C VIM quintile 1	2.228, 1.290–3.846	**0.004**
MCI-risk model including *APOE* genotypes		
Age	1.025, 1.003–1.049	**0.030**
Atherosclerosis × age	0.976, 0.958–0.995	**0.015**
*APOE* ε4 genotype × age (relative to non-ε4 genotypes)		
*APOE* ε4 heterozygote	1.004, 0.999–1.009	0.106
*APOE* ε4 homozygote	1.023, 1.013–1.034	**<0.001**
sex	1.451, 1.042–2.019	**0.027**
college	0.660, 0.441–0.986	**0.042**
HDL-C trajectory group (relative to group 3)		
1	3.571, 1.306–9.765	**0.013**
2	2.138, 0.823–5.556	0.119
Non-HDL-C VIM quintile 1	2.500, 1.512–4.133	**<0.001**

*P* < 0.05 in bold.

a The table presents the results of logistic regression models of AD- and MCI-risk in the AD and MCI case/control cohorts, respectively, developed as described in the text. Models with polygenic risk scores as covariates (*case ∼ covariates + lipid-trajectory groups + 10 genetic PCs + polygenic risk scores*) used only individuals with EUR ancestry, as most of these scores were developed in European populations, while models with *APOE* genotypes as covariates (*case ∼ covariates + lipid-trajectory groups + APOE* genotypes) used the full case/control cohorts.

One set of models included PRS (*PRS models*) previously developed using summary statistics from GWAS of AD or lipid levels; this set of models only used data from individuals with EUR ancestry. In the PRS model for AD, increased risk was associated with lower HDL-C trajectory groups, the lowest VIM quintile for TC, increasing age, cerebrovascular disease × age, higher AD PRS score, and higher TC PRS score, whereas atherosclerosis × age and > 12 years of education were associated with reduced risk (Fig. 4A). In the PRS model for MCI, increased risk was associated with lower HDL-C trajectory groups, the lowest VIM quintile for non-HDL-C, and higher AD PRS score, whereas more than 12 years of education and atherosclerosis × age were associated with decreased risk (Fig. 4B). The predictive performance of models incorporating only base covariates or those including base covariates and PRS improved for both AD and MCI when the lipid trajectory groups were included (Fig. 4C, D).

A second set of models that evaluated the contribution of *APOE* status (*APOE models*) included all ancestries (supplemental Table S3). In the *APOE* model for AD, increased risk was associated with the lower HDL-C trajectory groups, lowest VIM quintile of TC, and *APOE-*ε4 × age. Having more than 12 years of education was associated with decreased risk, and atherosclerosis × age had no significant effect (Fig. 4E). In both models, the best fit was obtained if a history of hypertension was included, although this association did not reach significance. The *APOE* model for MCI showed similar associations, but only the lowest HDL-C level trajectory group was associated with increased risk. An increased risk of MCI with *APOE-*ε4 × age only reached significance for *APOE-*ε4 homozygotes. Female sex was also associated with increased risk for MCI (Fig. 4F). The predictive performance of models incorporating only base covariates or base covariates with APOE status improved for both AD and MCI when lipid trajectory groups were included (Fig. 4G, H).

In both *APOE* and PRS models, the odds ratios and CIs associated with HDL-C trajectory groups 1 and 2 are stated relative to HDL-C trajectory group 3 (Table 3 and Fig. 4A, B, E, F). About 10% of the MCI case/control cohort and about 15% of the AD case/control cohort was assigned to trajectory group 3 (supplemental Table S4 and Fig. 2B, G). The relatively wide CIs for the odds ratios associated with risk of MCI or AD in HDL-C trajectory groups 1 and 2, which reflect uncertainty, arise in part because of the comparatively smaller size of the reference group (HDL-C trajectory group 3), in each case/control cohort, which reflects how group-based trajectory models identified latent groups in these cohorts.

### Relationships predicted by the risk models

**Fig. 5:**
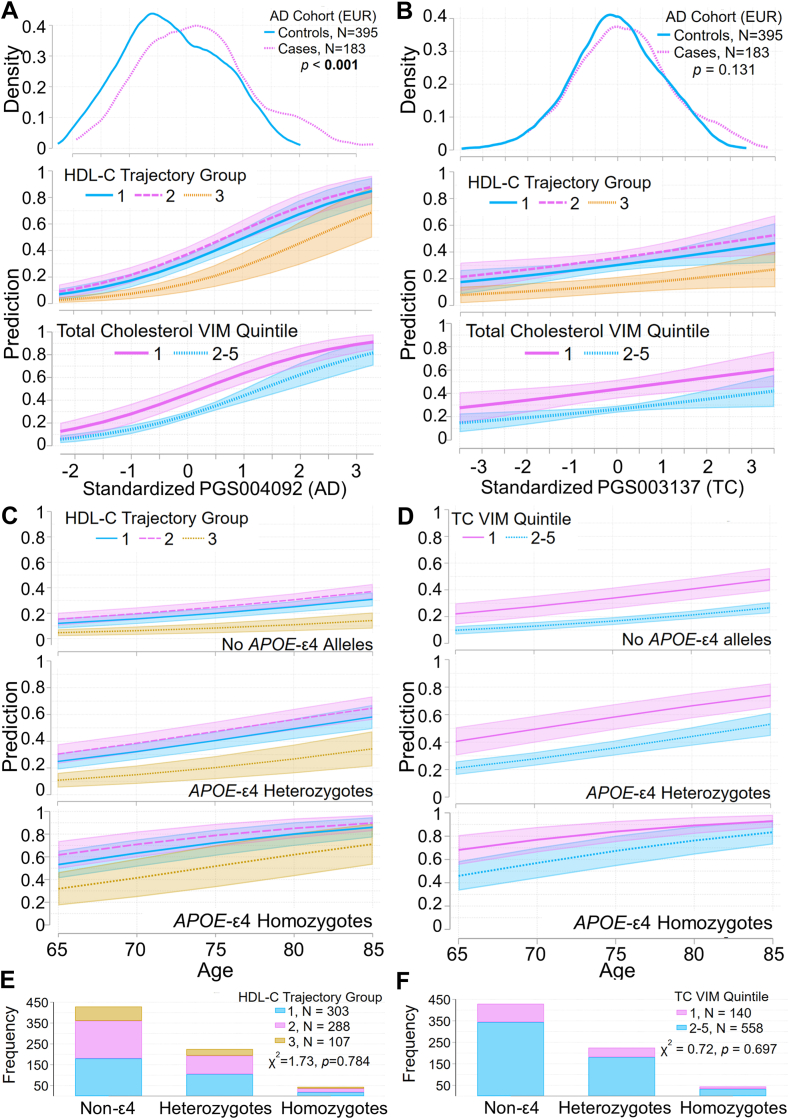
Contributions of HDL-C trajectory group and lowest VIM quintile for total cholesterol to AD risk, relative to genetic risk factors. The covariate-adjusted AD-risk model including polygenic risk scores summarized in Figure 4A and Table 3 was used to estimate contributions to AD risk relative to polygenic risk scores for AD and total cholesterol. and relative to *APOE*-ε4 genotype. Panel (A) shows the predictive margins for HDL-C trajectory groups and for quintiles 1 (lowest) and 2–5 of total-cholesterol VIM over a range of values of PGS004092 (AD) at age 75 and at mean values of other covariates. Panel (B) shows the predictive margins for these trajectory groups and quintiles over a range of values of PGS003137 (total cholesterol) at age 75 and at mean values of other covariates. Lines show the predicted AD risk; shaded areas indicate 95% confidence intervals. For comparison, kernel-density plots obtained using an Epanechnikov kernel function illustrate the distribution of each polygenic risk score in AD cases and matched controls. A Kolmogorov-Smirnov test evaluated the equality of the distribution of each polygenic risk score in cases and controls. The covariate-adjusted AD-risk model including *APOE* genotypes summarized in Figure 4E and Table 3 was used to estimate contributions to AD risk relative to *APOE*-ε4 genotype. Panel (C) shows the predictive margins for HDL-C trajectory groups over ages 65 to 85 and at mean values of other covariates in non-*APOE*-ε4 genotypes, *APOE*-ε4 heterozygotes, and *APOE*-ε4 homozygotes. Panel (D) shows the predictive margins for quintiles 1 and 2–5 of total-cholesterol VIM over ages 65 to 85 and at mean values of other covariates in these genotypes. Lines indicate predicted AD risk; shaded areas indicate 95% confidence intervals. Bar plots show the distribution of HDL-C trajectory groups (E) and total-cholesterol VIM quintile groups (F) across *APOE*-ε4 genotypes. A χ^2^-contingency test evaluated whether the distribution of three HDL-C trajectory groups or total-cholesterol VIM quintiles 1 versus 2–5 differed across *APOE*-ε4 genotypes. AD, Alzheimer's disease; VIM, variability independent of the mean.

Model-predicted relationships between AD risk and HDL-C trajectory group or VIM quintile for TC relative to genetic factors were visualized by plotting predicted margins for each model. Figure 5A, B shows the adjusted AD risk prediction with CIs for each lipid group for patients at age 75 over the range of PRS_AD_ (PGS004092) and PRS_TC_ (PGS003137) scores. The density distribution of each standardized PRS in cases and controls is shown for comparison. For individuals whose standardized PRS_AD_ scores lay in the interval [-1.2, 1.5] (∼10^th^ − 93^rd^ percentiles) or whose standardized PRS_TC_ scores lay in the interval [-1.7, 1.8] (∼fourth − 96^th^ percentiles), AD risk was lower in HDL-C trajectory group 3 (highest levels) than in HDL-C trajectory groups 1 and 2 (lower levels), and higher in TC VIM quintile 1 (least variability) than quintiles 2–5. For more extreme (higher or lower) PRS risk scores, the CIs of AD risk overlapped, suggesting that at very low or high levels of genetic AD risk, lipid status has no distinguishable effect.

Figure 5C, D shows the adjusted AD risk prediction with CIs for each lipid group over a range of ages for patients with no *APOE-*ε4 alleles, *APOE-*ε4 heterozygotes, and *APOE-*ε4 homozygotes. For AD models, HDL-C trajectory group 3 or TC VIM quintiles 2–5 were associated with lower risk in participants aged 64–85 with no *APOE-*ε4 alleles and in *APOE-*ε4 heterozygotes, but not for *APOE-*ε4 homozygotes. This suggests that the genetic risk conferred by *APOE-*ε4 in homozygotes, but not in heterozygotes, overrides risk associated with lipid-group membership. However, the wide, overlapping CIs for AD risk associated with lipid groups in *APOE-*ε4 homozygotes could reflect the small number of these individuals in this cohort. The distribution of *APOE-*ε4 genotypes is similar across HDL-C trajectory groups and the VIM quintiles of TC (Fig. 5E, F).

For MCI models, the predicted margins were also plotted (supplemental Fig. S3). Here, the CIs for MCI risk in the 5^th^ and 95^th^ percentiles of AD PRS scores in patients aged 65–85 were distinct, but both overlapped with the CIs of the 50^th^ percentile (supplemental Fig. S3A). Supplemental Fig. S3B, C shows the adjusted MCI risk prediction with CIs for each lipid group for patients at age 75 over the range of standardized PRS_AD_ (PGS004092). The density distribution of the standardized PRS in cases and controls is shown in supplemental Fig. S3D. While the CIs for MCI risk at age 75 in HDL-C trajectory group 2 (intermediate concentration) overlapped with those for trajectory groups 1 (highest concentration) and 3 (lowest concentration) across the range of PRS scores, these CIs were distinct for individuals in trajectory groups 1 and 3 whose scores were in the interval [-1, 1.5] (∼15^th^ − 95^th^ percentiles) (supplemental Fig. S3B). Furthermore, the CIs for MCI risk in the first and second-to-fifth non-HDL-C quintiles at age 75 did not overlap for scores near median (∼43^rd^ to 79^th^ percentile) values (supplemental Fig. S3C). This too suggests that the effect of lipid group membership is tempered by genetic AD risk: it has no distinguishable effect at very low or high levels of genetic AD risk.

The CIs for MCI risk by HDL-C trajectory group for each *APOE-*ε4 genotype in patients aged 50–85 overlapped (supplemental Fig. S4A, C, E). The CIs for MCI risk in non-HDL-C VIM quintile 1 and quintiles 2–5 did not overlap over the PRS_AD_ score range [−0.35, 0.1] (∼40^th^ − 80^th^ percentiles) in non-*APOE-*ε4 genotypes over age 64 (supplemental Fig. S4B), or in *APOE-*ε4 heterozygotes over age 66 (supplemental Fig. S4D); however, they did overlap in *APOE-*ε4 homozygotes in patients aged 50–85 (supplemental Fig. S4F), although, again, this may reflect the small number of *APOE-*ε4 homozygotes in this cohort. The distribution of *APOE-*ε4 genotypes was similar across HDL-C trajectory groups (supplemental Fig. S4G), and across non-HDL-C VIM quintiles 1 and 2–5 (supplemental Fig. S4H).

Taken together, these results indicate that the increased risk for both AD and MCI associated with lower HDL-C levels and low VIM for TC over the decade prior to symptom onset was somewhat independent of genetic and other risk factors.

## Discussion

In this study, we tested the hypothesis that routine blood-lipid measurements (TC, HDL-C, LDL-C, non-HDL-C, and triglycerides), obtained during the decade prior to cognitive-symptom onset, can inform risk prediction for AD and stable MCI. We studied clinically well-characterized, propensity-matched case/control cohorts and, in each cohort, assessed longitudinal correlates of lipid concentrations and grouped patients by their lipid trajectories and VIM of blood lipid concentrations. We then assessed whether group membership contributed to disease risk by developing risk models that evaluated the contributions of the longitudinal correlates, *APOE* genotype or PRS for AD and lipid levels, demographics, and medical history. In these models, groups in trajectories with lower HDL-C levels or with the lowest quintile of TC VIM were significantly associated with increased risk of AD and MCI. The inclusion of lipid-trajectory and VIM groups improved risk-model predictive performance.

### Lipid trajectories and VIM do not independently contribute to AD- and MCI-risk

Our results provide an important real-world perspective on aging-related cognitive outcomes that are influenced by lifestyle, environmental, cellular, and genetic factors that regulate lipid metabolism and lipid levels; genetic factors conferring risk of AD and MCI; and comorbidities associated with aging and cardiovascular health. Our aim was not to examine the mechanisms underlying development of AD or MCI, or the relationship between blood-lipid concentrations and lipid metabolism in the brain, but to obtain useful clinical insights that may inform risk prediction. Our models indicate that, in this patient population, lower HDL-C trajectories and the lowest quintile of TC VIM were associated with an increased risk of AD when controlling for other factors that contribute to AD risk: older age, cerebrovascular disease × age, higher PRS_AD_, and higher PRS_TC._ Lower risk was associated with atherosclerosis × age and more than 12 years of education. Some comorbidities previously associated with AD, such as diabetes, were not retained in our risk models, while some measures of cardiovascular health, such as atherosclerosis, appeared to be protective for AD. Though seemingly counterintuitive, this may suggest that medications used to treat conditions such as hypertension, atherosclerosis, or diabetes also lower the risk of AD, as suggested previously (10).

In this study, patients with the lowest quintile of TC VIM showed increased AD risk (OR [CI]: ∼2.5 [1.5–4.2]), and patients with the lowest quintile of non-HDL-C VIM showed increased risk of MCI (∼2.3 [1.3–4]) (Table 3). Cholesterol and non-HDL-C VIM are strongly positively correlated (*r* = 0.93 in each cohort), and the retention of VIM quintiles of different lipids in each model reflects that which gave the best model fit. In our study population, individuals with the lowest quintile of non-HDL-C or TC VIM also had the least use of cholesterol-lowering medication (supplemental Tables S6 and S7). Though medication use was not retained as a significant covariate in our risk models, less use of lipid-lowering medication (at matching age) may be indirectly related to increased AD- and MCI-risk in this population, which is consistent with meta-analyses suggesting an association between lipid-lowering therapy and all-cause dementia risk (80, 81).

In contrast to our findings, Moser *et al.* (38) found that the highest quintiles of TC and triglyceride VIM had an increased risk of incident AD/AD-related dementia (AD/ADRD), and Chung *et al.* (37) found that higher quartiles of TC VIM were associated with all-cause dementia and AD. Several important methodological differences might underlie these different findings: i) our study calculated VIM based on at least three lipid measurements obtained over the ten years immediately prior to first-symptom onset in cases or matching age in controls, in a clinically focused population. Moser *et al.* calculated VIM for a large community population (N = 11,571) based on at least three lipid measurements over a five-year period and evaluated risk of AD/ADRD over the following 12.9 years. Chung *et al.* calculated VIM using at least three lipid measurements for a very large cohort (N = 131,965; patients with prior diagnoses of stroke, dementia, or diabetes mellitus were excluded) over a prior five-year period and evaluated incident disease over a median follow-up of 8.4 years. ii) Our study evaluated only cases where AD and MCI were confirmed through longitudinal, multi-year follow-up, and we excluded non-AD dementia whereas the other studies evaluated incident AD/ADRD or AD/all-cause dementia by ICD code (33) or ICD-code and prescription of an antidementia medication based on a validated scale for assessment of cognitive dysfunction (32). iii) Our study evaluated the risk of AD or MCI using a logistic regression model with retrospective data on lipid trajectories and VIM during the decade prior to first-symptom onset. The previous reports evaluated risk of incident AD/ADRD or AD/all-cause dementia by analyzing survival free of AD/ADRD using Cox (38) or Fine-Gray (32) hazards regression models. Although the methodologies used in these two studies are not directly comparable with ours, the strength of our approach is that lipid levels were evaluated over a longer period and relative to the time of first symptom onset, and compared to matched controls from a clinically defined and longitudinally monitored, albeit smaller, population.

### High or low genetic risk may supersede lipid-associated risk

When we used our data to model disease risk, the risk associated with different lipid trajectory groups was indistinguishable in individuals with high (or low) PRS_AD_ and PRS_TC_, and in *APOE*-ε4 homozygotes (Fig. 5, supplemental Fig. S3, and S4). This may be partly explained by our sample size, since larger CIs were observed in *APOE*-ε4 homozygotes where estimates were based on relatively few individuals. However, under the ApoE cascade hypothesis (82), *APOE* genetic status impacts a cascade of events at the cellular and systems levels, leading to aging-related pathogenesis. *APOE*-ε4 homozygosity or higher PRS_AD_ (which includes the *APOE* region) or PRS_TC_ percentiles may be associated with impaired lipid metabolism, increased cellular stress, and dysregulation of intracellular trafficking, leading to perturbation of cellular lipid homeostasis. Individuals with these genetic factors might be expected to have AD or MCI risk that supersedes the effect different lipid-group memberships.

### Lipid trajectory group membership is not strongly related to quintile of lipid VIM

Though the mean lipid concentrations we calculated for a trajectory group were stable longitudinally (Fig. 2), lipid concentrations seen in individuals of a trajectory group could nonetheless vary over time. Indeed, no associations between the quintile of a lipid VIM and a lipid trajectory group retained significance after a Bonferroni correction for multiple tests (supplemental Tables S6–S10). Our analyses of the longitudinal correlates of lipid concentrations (Table 2) and associations with VIM quintile provide some insights into factors that contribute to an individual's trajectory group assignment and their VIM. Lipid PRSs were significant in analyses of the longitudinal correlations of lipid concentrations in these cohorts. This suggests that in addition to known confounders (e.g., age, sex, BMI, comorbidities, and lipid-lowering medication use) that were identified in the analyses of longitudinal correlates, genetic factors contribute to setting the mean lipid concentrations seen in each trajectory group. Our analyses support the hypothesis that lipid variation in individuals over time may be associated with adherence to lipid-lowering therapy or changes in diet or metabolism that in turn could be associated with aging, onset of a comorbidity or cognitive dysfunction, or other factors that we did not assess, such as lifestyle changes.

### Strengths and limitations

One of the strengths of this study is that DSM-IV criteria were used to diagnose (cases) and exclude patients with dementias that were not AD, mixed AD and VaD, or MCI. While more stringent diagnostic criteria for some dementias could have been used (e.g., for VaD (83)), this would likely exclude fewer patients. Patients were followed longitudinally by neurologists using SCDS toolkits designed for standardized recording of clinical data on neurological disorders at point of care, thus reflecting disease course more accurately. Mean follow-up was 6.5 years for AD and 7.8 years for MCI, providing confidence in the clinical status of patients in our study groups. Our approach also allowed us to reliably identify the year of first symptom onset, which cannot be readily inferred when ICD codes are used as a proxy for diagnosis, and which was critical to retrospectively evaluating lipid data stored in patient EHRs. We were also able to identify matched controls without neurodegenerative diseases.

Recently, efforts have been made to develop a biological definition of AD using a combination of biomarkers that include Aβ proteinopathy, assessed from cerebrospinal fluid or plasma Aβ assays or amyloid PET imaging, and phosphorylated and secreted AD tau, assessed from cerebrospinal fluid or plasma assays for p-tau217, p-tau181, and p-tau231 (84). Our approach complements the efforts to define a biological basis for AD. It would be valuable to longitudinally assess these biomarkers, which can be used to define the biological characteristics and onset of AD, as well as biomarkers identified using lipidomic approaches (e.g., (7, 44, 45, 47, 85)) during aging in a clinically well-characterized, diverse, and genotyped population. This would validate estimates of risk associated with specific types or sets of lipids and ApoA1-or ApoB-containing lipoproteins identified in cross-sectional studies and contribute to a mechanistic understanding of AD- or MCI-risk.

A second strength of this study is the use of group-based trajectory models to identify groups with different longitudinal lipid trajectories. The model-fit statistics for these models provide confidence for group assignments within each case/control cohort. In the AD case/control cohort, lower mean HDL-C concentrations (∼40 mg/dl) in trajectory group 1 were associated with increased AD risk relative to the higher mean concentrations (>80 mg/dl) in trajectory group 3 (OR [CI] ∼3.8 [2.0–7.3]; compare Fig. 2B and supplemental Table S5).

A third strength is that we considered potential confounders when we developed our models, so that risk associated with lipid groups was evaluated relative to the longitudinal correlates of lipid concentrations, comorbidities, and patient medical history. For example, we evaluated atherosclerosis, which has been associated with blood lipid concentrations and VaD, as a covariate.

Limitations of this study include the sample size, the interval over which we were able to retrospectively obtain blood lipid measurements, which was limited by available EHR data, and the methods used to assess lipid concentrations that were initially obtained for clinical purposes—for example, we were unable to assess levels of intermediate-density lipoprotein cholesterol, VLDL-C, or other more specific lipoprotein subclasses. The prospective study described above would overcome these limitations.

Both the size of our case/control cohorts (AD, N = 698; MCI, N = 551) and our requirement that at least 5% of each cohort be present in the smallest group constrained the number of trajectory groups. Indeed, additional lipid-level trajectory groups were identified in a study with a larger cohort (65). For this reason, we caution against extending the mean lipid values from our lipid trajectory groups to other cohorts.

### Future Directions

These results extend our understanding of the complex set of relationships between variation in blood-lipid levels and comorbidities associated with AD and MCI. It will be important to further clarify the relative contributions of genetic factors and blood-lipid profiles, specifically to risk of AD or MCI, and undertake studies to dissect the mechanism(s) that underlie the risk relationships.

Several pragmatic questions arise from this work. First, whether risk of disease, more specifically genetic risk, can be reduced by modifying blood-lipid levels, particularly in patients whose lipid PRS scores indicate they are at greater genetic risk for lipid trajectories that increase their risk of AD or MCI. Second, whether disease trajectory after diagnosis can be altered by modifying lipid levels, including by managing other comorbidities associated with high or low blood lipid levels. Addressing such questions requires detailed, clinical characterization of patients over time using point-of-care SCDS tools, such as the one used here. This study thus supports longitudinal documentation of clinical changes during the evolution of disease processes, which can then be evaluated in the context of genetic risk factors as well as aging, comorbidity, and treatment effects.

## Data Availability

Summary statistics are available from the corresponding author upon reasonable request, following institutional approval. Since the data collected for this study include protected health information subject to the regulations of the Health Insurance Portability and Accountability Act and an informed consent process, requests to access the data set from qualified researchers with training in protocols to maintain human subject confidentiality will be considered, subject to a data use agreement.

## Conflict of interest

The authors declare that they have no conflicts of interest with the contents of this article.
