# Supplementary material for: Lipid trajectories improve risk models for Alzheimer’s disease and mild cognitive impairment
Source: J Lipid Res. 2024 Nov 23;66(1):100714. doi: 10.1016/j.jlr.2024.100714 (PMC11731482; doi:10.1016/j.jlr.2024.100714)

Item **Page**

Table S1 Participants by DodoNA-project study 2

Table S2 DodoNA memory-toolkit data elements 3

Table S3 Ancestry information in AD and MCI case/control cohorts 6

Table S4 Groups identified in group-based trajectory models for lipid levels in the AD and MCI case/control cohorts 11

Table S5 Chi-square tests of association of MCI and AD with lipid trajectory groups 13

Table S6 Cohort characteristics by quintile of total-cholesterol variability independent of the mean (VIM) 14

Table S7 Cohort characteristics by quintile of non-HDL-C VIM 19

Table S8 Cohort characteristics by quintile of HDL-C VIM 24

Table S9 Cohort characteristics by quintile of LDL-C VIM 29

Table S10 Cohort characteristics by quintile of ln(triglycerides) VIM 34

Figure S1 Age at first cognitive symptom 39

Figure S2 Lipid measurements in the AD and MCI case-control cohorts 40

Figure S3 Contributions of HDL-C trajectory groups and the lowest quintile of total non-HDL-C VIM to MCI risk, relative to an AD polygenic risk score 41

Figure S4 Contributions of HDL-C trajectory groups and the lowest quintile of non-HDL-C VIM to MCI risk, relative to *APOE*-ε4 genotype 42

Table S1. Participants by DodoNA-project study.

DodoNA Study	Total Enrollment	Genotyped	Memory Toolkit Use[a]	AD Cases	AD Controls[b]	MCI Cases	MCI Controls[b]	Citations
**Brain health**	830	537	24	5	67	4	61	(53)
**Neuro-oncology**	447	316	6	–	30	–	22	(54)
**Epilepsy**	1,065	758	14	1	29	–	31	(55,56)
**Headache (migraine)**	2,585	2,012	13	–	51	–	38	(57)
**Mild traumatic brain injury**	798	584	10	1	20	–	18	(58)
**Memory**	1,271	1,004	1,004	208	–	109	-	(59)
**Multiple sclerosis**	739	571	3	–	13	–	5	(60)
**Neuromuscular (polyneuropathy)**	1,178	759	11	–	62	2	74	(61,62)
**Parkinson's disease**	1,070	956	12	–	–	–	-	(63,64)
**Sleep (restless leg syndrome)**	1,158	897	20	–	1	–	2	(65)
**Stroke**	1,357	1,015	31	–	206	1	184	(66)
**Total**	12,498	9,409	1,148	215	483	116	435	
**EUR ancestry** (in cases and controls)	–	–	–	183	395	88	345	

[a]This column gives the numbers of genotyped patients who were evaluated using the memory SCDS toolkit. Cases were identified from patients who were followed using the memory SCDS toolkit and who satisfied study inclusion and exclusion criteria.

[b]Controls were selected from patients enrolled in the DodoNA project and satisfied study inclusion and exclusion criteria. Individuals enrolled in the DodoNA Parkinson's disease study and patients with neurodegenerative disease or cognitive impairment were specifically excluded.

Table S2. DodoNA memory-toolkit data elements.[a]

Data Element	Recorded As
**History**	
Encounter date	date
Birth date	
Sex	M/F
Allergies/Contraindications	details
Tobacco Use	former/daily/some days/unknown, smokeless, passive exposure
Alcohol use	yes/not currently/never, drinks per week
Drug use	yes/not currently/never, type, per week
Sexual activity	Active/ever active, birth control/protection, partners
**Social History**	
Occupational history	details
Marital status	
Children	
Years of education	
Social drivers of health	alcohol use, food insecurity, stress, housing stability, interpersonal safety, financial resource strain, transportation needs, intimae partner violence, utilities, health literacy
**Demographics**	
nature of visit	initial, interval, annual
domicile	home, nursing home, religious community, other
self-repoted race/ethnicity	details
**Vitals**	
Blood pressure (systolic, diastolic)	mm Hg
Temperature	^{0}F
Pulse	Pulse rate
Respiration	Respiration rate
Weight	kg
Height	m
Body mass index (BMI)	kg/m^2
SpO2	percent
Pain score / location	details
**Medical History**	
Neurological	details
Sleep related	
Cardiovascular	
Autoimmune disease	
Behavioral	
Hormonal	
Medication	
B12 deficiency	
Folate deficiency	
Heart disease	

Liver failure	
Renal disease	
Normal pressure hydrocephalus	
Deafness	
Glaucoma	
Macular degeneration	
Other	
**Surgical history**	
**Family history**	
Allergies, arthritis, blood disease, cancer, dermatological, diabetes, endocrine, ENT, hypertension, neurological, OB/GYNE, osteoporosis, psychiatry, pulmonary/lung, smoking, stroke, thyroid disease, vision, other	Presence / absence of each in enumerated first-degree relatives
**Objective Test Scores**	
Barthel index	informant and individual question and total scores
FAQ	
Geriatric Depression Scale	
Short test of mental status	individual question and total scores
Montreal cognitive assessment test	
Unified Parkinson's Disease Rating Scale	
**Initial Symptoms**	
Informant	patient, spouse, sibling, offspring, friend, other
Year of onset	year, don't know
Initial symptoms	memory difficulty, word-finding difficulty, vision change, other cognitive difficulty, behavioral change, gait disorder/falls, apraxia, involuntary movements, urinary incontinence, other (specify)
Mode of onset	don't know, abrupt onset, insidious onset
Course of symptoms	don't know, slow progression, rapid progression, stepwise progression, static, improving, resolved
**Current Symptoms**	
Memory	none, recent ("short term") memory loss, repeating questions/comments, losing, misplacing items, remote memory loss, semantic memory loss, procedural memory loss
Language	none, word finding difficulties, name recall, reading/writing, gloval paraphasias, phenomic paraphasias
Visual Spatial	none, facial recognition deficit, visual spatial deficit, navigation difficulties/getting lost
Attention/Executive	non, disorientation, organization difficulties/problem solving deficit, distractibility/inattention, dyscalculia, judgment impaired

Gait disorder	non, shiffling, uses cane, uses walker, uses wheelchair, decreased balance, other (specify)
Falls	none, mechanical, orthostatic lightheadedness, assicated injuries, other (specify)
Type of involuntary movements	non, tremor, myoclonus, chorea, other (specify)
Apraxia	none, right side predominant, left side predominant, no predominance
Behavioral and psychological	none, depression/dysphoria, apathy/indifference, anxiety, elation/euphoria, irritability/lability, agitation/aggression, disinhibition, psychomotor disturbance (perseveration, obsessive-compulsive), hoarding, hallucinations (visual, auditory), delusions (persecution, misidentification, reduplicatoin), dleep disturbance, appetitie, other (specify)
Additional symptoms	non, seizures, anosima, autonomic, wandering, other (specify)
**Hospital visits**	
Emergency department visits	past year (yes/no)
Hospitalizations	
**Diagnostic studies**	CT head, MRI brain scanning, PET glucose, PET amyloid, DaTscan, EEG, neuropsychology, polysomnography, genetic testing (*APOE*, *APP*, *PS1*, *PS2*, other), CSF amyloid/tau ratio
**Clinical Impression**	
Cognitive impairment	Yes / No
Meets DSM-IV criteria for Mild Cognitive Impairment (MCI)	
Subtype of MCI	Amnestic type, single domain / Non-amnestic type, single domain / Amnestic type, multi-domain / non-amnestic type, multi-domain
Meets DSM-IV criteria for dementia	Yes / No
Alzheimer's disease	
Vascular dementia	
Lewy body dementia	
Frontotemporal dementia	
Primary progressive aphasia	
Other dementia / other cause	detail of diagnosis: parkinsonism, stroke, normal pressure hydrocephalus, cortical basal degeneration, progressive supranuclear palsy, stroke, alcohol use, end-stage-renal disease, subdural hematoma, B12 deficiency, psychiatric disorder, radiation therapy, etc.
Functional Assessment Staging Tool (FAST)	1, 2, 3, 4, 5, 6a-e, 7a-f

[a]Demographics, family history and AAO were collected only at study enrollment. All other data elements were collected at study enrollment and at each annual follow-up.

Table S3. Ancestry information in AD and MCI case/control cohorts.

AD Case/Control Cohort									
			Ancestry						
			African	Admixed American	East Asian	European	Greater Middle East	South Asian	Unknown
**N**		controls	9 (1.9)	0 (0)	16 (3.3)	395 (81.8)	12 (2.5)	12 (2.5)	39 (8.1)
		cases	3 (1.4)	0 (0)	6 (2.8)	183 (85.1)	3 (1.4)	2 (0.9)	18 (8.4)
		total	12 (1.7)	0 (0)	22 (3.8)	578 (96.7)	15 (2.5)	14 (2.3)	57 (9.5)
**Age at first lipid measurement, md (r)**		controls	57 (42-79)	–	68 (50-83)	68 (43-89)	74 (60-85)	68 (51-82)	73 (47-85)
		cases	74 (59-76)	–	66 (60-82)	72 (46-87)	62 (60-71)	69 (63-75)	77 (60-84)
**Match age, controls onset age, cases; md(r)**		controls	70 (58-86)	–	74 (58-87)	77 (52-94)	81 (72-89)	77 (61-89)	81 (52-93)
		cases	78 (65-78)	–	76 (66-89)	79 (52-94)	72 (70-75)	75 (71-79)	83 (67-91)
**Age at last follow-up/death, md (r)**		controls	69 (52-95)	–	77 (65-101)	80 (55-103)	84 (70-99)	79 (75-92)	83 (60-98)
		cases	85 (70-92)	–	83 (74-93)	86 (59-98)	78 (77-88)	79 (77-81)	90 (70-100)
**BMI, md (r)**		controls	29 (24-34)	–	24 (16-34)	26 (18-40)	25 (23-30)	25 (20-32)	26 (18-37)
		cases	29 (26-29)	–	26 (22-27)	25 (16-39)	22 (22-25)	24 (22-26)	26 (20-32)
**Female**		controls	5 (1)	–	10 (2.1)	206 (42.7)	4 (0.8)	3 (0.6)	15 (3.1)
		cases	3 (1.4)	–	3 (1.4)	93 (43.3)	2 (0.9)	2 (0.9)	9 (4.2)
**Blood-cholesterol lowering medications used**		controls	2 (0.4)	–	15 (3.1)	297 (61.5)	11 (2.3)	10 (2.1)	30 (6.2)
		cases	1 (0.5)	–	5 (2.3)	133 (61.9)	1 (0.5)	2 (0.9)	16 (7.4)
***APOE*-ε2 alleles**	**0**	controls	7 (1.4)	–	15 (3.1)	345 (71.4)	11 (2.3)	9 (1.9)	32 (6.6)
		cases	3 (1.4)	–	4 (1.9)	168 (78.1)	3 (1.4)	2 (0.9)	18 (8.4)
	**1**	controls	2 (0.4)	–	1 (0.2)	47 (9.7)	0 (0)	3 (0.6)	7 (1.4)
		cases	0 (0)	–	2 (0.9)	15 (7)	0 (0)	0 (0)	0 (0)
	**2**	controls	0 (0)	–	0 (0)	3 (0.6)	1 (0.2)	0 (0)	0 (0)
		cases	0 (0)	–	0 (0)	0 (0)	0 (0)	0 (0)	0 (0)
	**0**	controls	4 (0.8)	–	10 (2.1)	264 (54.7)	11 (2.3)	10 (2.1)	28 (5.8)

		cases	1 (0.5)	–	5 (2.3)	84 (39.1)	1 (0.5)	2 (0.9)	9 (4.2)
***APOE*-ε4 alleles**	**1**	controls	4 (0.8)	–	5 (1)	116 (24)	1 (0.2)	2 (0.4)	11 (2.3)
		cases	2 (0.9)	–	0 (0)	73 (34)	2 (0.9)	0 (0)	9 (4.2)
	**2**	controls	1 (0.2)	–	1 (0.2)	15 (3.1)	0 (0)	0 (0)	0 (0)
		cases	0 (0)	–	1 (0.5)	26 (12.1)	0 (0)	0 (0)	0 (0)
**Years of education**	**< 12**	controls	0 (0)	–	0 (0)	14 (2.9)	0 (0)	0 (0)	0 (0)
		cases	0 (0)	–	0 (0)	4 (1.9)	0 (0)	1 (0.5)	0 (0)
	**12**	controls	5 (1)	–	4 (0.8)	46 (9.5)	1 (0.2)	0 (0)	2 (0.4)
		cases	1 (0.5)	–	2 (0.9)	40 (18.6)	0 (0)	0 (0)	2 (0.9)
	**12–15**	controls	1 (0.2)	–	2 (0.4)	69 (14.3)	3 (0.6)	1 (0.2)	5 (1)
		cases	1 (0.5)	–	0 (0)	27 (12.6)	0 (0)	0 (0)	4 (1.9)
	**≥ 16**	controls	3 (0.6)	–	10 (2.1)	266 (55.1)	8 (1.7)	11 (2.3)	32 (6.6)
		cases	1 (0.5)	–	4 (1.9)	112 (52.1)	3 (1.4)	1 (0.5)	12 (5.6)
**Smoking**		controls	2 (0.4)	–	0 (0)	7 (1.4)	0 (0)	1 (0.2)	1 (0.2)
		cases	0 (0)	–	0 (0)	1 (0.5)	0 (0)	0 (0)	0 (0)
**Moderate-to-heavy alcohol use**		controls	0 (0)	–	0 (0)	51 (10.6)	1 (0.2)	0 (0)	0 (0)
		cases	0 (0)	–	0 (0)	19 (8.8)	0 (0)	0 (0)	2 (0.9)
**Hypertension**		controls	9 (1.9)	–	15 (3.1)	288 (59.6)	11 (2.3)	9 (1.9)	32 (6.6)
		cases	3 (1.4)	–	5 (2.3)	130 (60.5)	0 (0)	2 (0.9)	16 (7.4)
**Atherosclerosis**		controls	1 (0.2)	–	0 (0)	30 (6.2)	1 (0.2)	1 (0.2)	2 (0.4)
		cases	0 (0)	–	0 (0)	7 (3.3)	0 (0)	1 (0.5)	2 (0.9)
**Cerebrovascular disease**		controls	1 (0.2)	–	2 (0.4)	70 (14.5)	1 (0.2)	2 (0.4)	7 (1.4)
		cases	1 (0.5)	–	1 (0.5)	48 (22.3)	1 (0.5)	0 (0)	5 (2.3)
**Diabetes**		controls	1 (0.2)	–	11 (2.3)	89 (18.4)	3 (0.6)	5 (1)	14 (2.9)
		cases	1 (0.5)	–	3 (1.4)	45 (20.9)	0 (0)	2 (0.9)	6 (2.8)
**Ischemic heart disease or myocardial infarction**		controls	2 (0.4)	–	5 (1)	124 (25.7)	9 (1.9)	5 (1)	17 (3.5)
		cases	0 (0)	–	2 (0.9)	52 (24.2)	0 (0)	1 (0.5)	6 (2.8)

**Malignant neoplasm**		controls	4 (0.8)	–	1 (0.2)	182 (37.7)	4 (0.8)	0 (0)	14 (2.9)
		cases	1 (0.5)	–	1 (0.5)	66 (30.7)	1 (0.5)	0 (0)	6 (2.8)
**Race**	Caucasian	controls	0 (0)	–	1 (0.2)	392 (81.2)	11 (2.3)	4 (0.8)	34 (7)
		cases	0 (0)	–	1 (0.5)	183 (85.1)	3 (1.4)	0 (0)	16 (7.4)
	Black/African American	controls	9 (1.9)	–	0 (0)	0 (0)	1 (0.2)	0 (0)	5 (1)
		cases	3 (1.4)	–	0 (0)	0 (0)	0 (0)	0 (0)	2 (0.9)
	Asian	controls	0 (0)	–	15 (3.1)	0 (0)	0 (0)	8 (1.7)	0 (0)
		cases	0 (0)	–	5 (2.3)	0 (0)	0 (0)	1 (0.5)	0 (0)
	Other	controls	0 (0)	–	0 (0)	3 (0.6)	0 (0)	0 (0)	0 (0)
		cases	0 (0)	–	0 (0)	0 (0)	0 (0)	1 (0.5)	0 (0)

MCI Case/Control Cohort

		**Ancestry**						
		**African**	**Admixed American**	**East Asian**	**European**	**Greater Middle East**	**South Asian**	**Unknown**
**N**	controls	7 (1.6)	2 (0.5)	16 (3.7)	345 (79.3)	14 (3.2)	13 (3)	38 (8.7)
	cases	2 (1.7)	2 (1.7)	4 (3.4)	88 (75.9)	4 (3.4)	1 (0.9)	15 (12.9)
	total	9 (1.6)	4 (0.7)	20 (3.6)	443 (78.6)	18 (3.3)	14 (1.2)	53 (9.6)
**Age at first lipid measurement, md (r)**	controls	68 (67-83)	49 (49)	65 (58-83)	68 (41-89)	71 (57-85)	65 (53-82)	68 (47-83)
	cases	72 (67-77)	64 (62-67)	80 (68-88)	71 (46-92)	72 (62-75)	67	73 (46-81)
**Match age, controls onset age, cases; md(r)**	controls	78 (75-93)	59 (59)	72 (68-92)	77 (53-94)	78 (66-89)	74 (59-87)	77 (56-93)
	cases	78 (75-82)	71 (71-72)	89 (75-93)	77 (51-98)	78 (68-81)	72	79 (55-86)
**Age at last follow-up/death, md (r)**	controls	80 (76-95)	58 (52-64)	74 (67-101)	79 (53-103)	80 (66-99)	79 (59-92)	81 (64-98)
	cases	85 (82-89)	78 (78)	97 (81-99)	84 (58-104)	86 (77-89)	85	86 (64-96)
**BMI, md (r)**	controls	27 (24-44)	28 (28)	25 (21-35)	27 (16-52)	26 (22-36)	25 (21-38)	27 (19-36)
	cases	33(28-38)	37 (33-40)	25 (24-26)	27 (16-41)	28 (22-36)	21	26 (16-35)
**Female**	controls	2 (0.5)	2 (0.5)	9 (2.1)	167 (38.4)	5 (1.1)	4 (0.9)	13 (3)
	cases	0 (0)	2 (1.7)	2 (1.7)	45 (38.8)	2 (1.7)	0 (0)	7 (6)

**Blood-cholesterol lowering medications used**		controls	4 (0.9)	0 (0)	14 (3.2)	274 (63)	13 (3)	12 (2.8)	31 (7.1)
		cases	2 (1.7)	2 (1.7)	3 (2.6)	64 (55.2)	4 (3.4)	1 (0.9)	13 (11.2)
***APOE*-ε2 alleles**	**0**	controls	4 (0.9)	2 (0.5)	16 (3.7)	306 (70.3)	12 (2.8)	9 (2.1)	32 (7.4)
		cases	2 (1.7)	2 (1.7)	3 (2.6)	77 (66.4)	3 (2.6)	0 (0)	13 (11.2)
	**1**	controls	3 (0.7)	0 (0)	0 (0)	39 (9)	2 (0.5)	4 (0.9)	5 (1.1)
		cases	0 (0)	0 (0)	1 (0.9)	10 (8.6)	1 (0.9)	1 (0.9)	2 (1.7)
	**2**	controls	0 (0)	0 (0)	0 (0)	0 (0)	0 (0)	0 (0)	1 (0.2)
		cases	0 (0)	0 (0)	0 (0)	1 (0.9)	0 (0)	0 (0)	0 (0)
***APOE*-ε4 alleles**	**0**	controls	3 (0.7)	2 (0.5)	15 (3.4)	246 (56.6)	13 (3)	11 (2.5)	26 (6)
		cases	2 (1.7)	1 (0.9)	4 (3.4)	52 (44.8)	3 (2.6)	1 (0.9)	10 (8.6)
	**1**	controls	4 (0.9)	0 (0)	1 (0.2)	93 (21.4)	0 (0)	2 (0.5)	11 (2.5)
		cases	0 (0)	1 (0.9)	0 (0)	29 (25)	1 (0.9)	0 (0)	5 (4.3)
	**2**	controls	0 (0)	0 (0)	0 (0)	6 (1.4)	1 (0.2)	0 (0)	1 (0.2)
		cases	0 (0)	0 (0)	0 (0)	7 (6)	0 (0)	0 (0)	0 (0)
**Years of education**	**< 12**	controls	0 (0)	0 (0)	0 (0)	8 (1.8)	1 (0.2)	0 (0)	0 (0)
		cases	1 (0.9)	0 (0)	0 (0)	1 (0.9)	0 (0)	0 (0)	1 (0.9)
	**12**	controls	4 (0.9)	2 (0.5)	2 (0.5)	42 (9.7)	1 (0.2)	2 (0.5)	3 (0.7)
		cases	0 (0)	0 (0)	1 (0.9)	19 (16.4)	1 (0.9)	0 (0)	1 (0.9)
	**12–15**	controls	2 (0.5)	0 (0)	4 (0.9)	67 (15.4)	3 (0.7)	3 (0.7)	6 (1.4)
		cases	1 (0.9)	1 (0.9)	0 (0)	16 (13.8)	1 (0.9)	0 (0)	1 (0.9)
	**≥ 16**	controls	1 (0.2)	0 (0)	10 (2.3)	228 (52.4)	9 (2.1)	8 (1.8)	29 (6.7)
		cases	0 (0)	1 (0.9)	3 (2.6)	52 (44.8)	2 (1.7)	1 (0.9)	12 (10.3)
**Smoking**		controls	2 (0.5)	0 (0)	0 (0)	6 (1.4)	0 (0)	0 (0)	1 (0.2)
		cases	0 (0)	0 (0)	0 (0)	3 (2.6)	0 (0)	0 (0)	0 (0)
**Moderate-to-heavy alcohol use**		controls	0 (0)	0 (0)	0 (0)	48 (11)	1 (0.2)	0 (0)	1 (0.2)
		cases	0 (0)	0 (0)	0 (0)	11 (9.5)	2 (1.7)	0 (0)	0 (0)
**Hypertension**		controls	7 (1.6)	1 (0.2)	14 (3.2)	256 (58.9)	13 (3)	11 (2.5)	32 (7.4)

		cases	2 (1.7)	2 (1.7)	4 (3.4)	70 (60.3)	3 (2.6)	1 (0.9)	11 (9.5)
**Atherosclerosis**		controls	2 (0.5)	0 (0)	0 (0)	28 (6.4)	3 (0.7)	1 (0.2)	1 (0.2)
		cases	0 (0)	0 (0)	0 (0)	2 (1.7)	0 (0)	0 (0)	0 (0)
**Cerebrovascular disease**		controls	0 (0)	0 (0)	3 (0.7)	58 (13.3)	2 (0.5)	1 (0.2)	7 (1.6)
		cases	0 (0)	0 (0)	1 (0.9)	18 (15.5)	1 (0.9)	0 (0)	3 (2.6)
**Diabetes**		controls	3 (0.7)	1 (0.2)	10 (2.3)	95 (21.8)	4 (0.9)	3 (0.7)	10 (2.3)
		cases	2 (1.7)	1 (0.9)	2 (1.7)	24 (20.7)	1 (0.9)	1 (0.9)	5 (4.3)
**Ischemic heart disease or myocardial infarction**		controls	2 (0.5)	0 (0)	3 (0.7)	128 (29.4)	8 (1.8)	5 (1.1)	19 (4.4)
		cases	0 (0)	0 (0)	0 (0)	28 (24.1)	3 (2.6)	0 (0)	7 (6)
**Malignant neoplasm**		controls	5 (1.1)	0 (0)	3 (0.7)	162 (37.2)	4 (0.9)	2 (0.5)	12 (2.8)
		cases	2 (1.7)	1 (0.9)	1 (0.9)	33 (28.4)	1 (0.9)	0 (0)	7 (6)
**Race**	Caucasian	controls	0 (0)	0 (0)	1 (0.2)	344 (79.1)	13 (3)	3 (0.7)	26 (6)
		cases	0 (0)	0 (0)	0 (0)	88 (75.9)	4 (3.4)	0 (0)	15 (12.9)
	Black/African American	controls	7 (1.6)	0 (0)	0 (0)	0 (0)	1 (0.2)	1 (0.2)	1 (0.2)
		cases	2 (1.7)	0 (0)	0 (0)	0 (0)	0 (0)	0 (0)	0 (0)
	Asian	controls	0 (0)	0 (0)	15 (3.4)	0 (0)	0 (0)	9 (2.1)	0 (0)
		cases	0 (0)	0 (0)	4 (3.4)	0 (0)	0 (0)	1 (0.9)	0 (0)
	Other	controls	0 (0)	2 (0.5)	0 (0)	1 (0.2)	0 (0)	0 (0)	1 (0.2)
		cases	0 (0)	2 (1.7)	0 (0)	0 (0)	0 (0)	0 (0)	0 (0)

[a]Unless otherwise specified, the table reports patient information at the age of first-symptom onset in cases and at the age of match in controls. Entries are N (percent of the total number of cases or percent the total number of controls). Ancestry was determined during genotype imputation using the TOPMED imputation server. Abbreviations: md, median; r, range; body mass index, BMI.

Table S4. Groups identified in group-based trajectory models for lipid levels in the AD and MCI case/control cohorts.

Lipid	Group Attribute	MCI case/control cohort, N = 551			AD case/control cohort, N = 698		
		Group 1	Group 2	Group 3	Group 1	Group 2	Group 3
Total Cholesterol	N (% of total)	154 (27.9)	282 (51.2)	115 (20.9)	319 (45.7)	305 (43.7)	74 (10.6)
	Fit[a]	linear	linear	linear	linear	linear	quadratic
	Average posterior probability	0.951	0.938	0.959	0.968	0.955	0.951
	Odds of correct classification[b]	50.5	14.6	90.0	35.7	27.1	133.3
	Observed probability[c]	0.293	0.497	0.210	0.452	0.437	0.111
HDL-C	N (% of total)	306 (55.5)	192 (34.8)	53 (9.6)	303 (43.4)	288 (41.3)	107 (15.3)
	Fit[a]	linear	linear	quadratic	linear	quadratic	quartic
	Average posterior probability	0.991	0.987	0.985	0.990	0.982	0.980
	Odds of correct classification[b]	84.5	148.1	627.3	125.6	79.6	264.3
	Observed probability[c]	0.553	0.350	.097	0.439	0.409	0.152
Non-HDL-C	N (% of total)	189 (34.3)	284 (51.5)	78 (14.2)	315 (45.1)	343 (49.1)	40 (5.7)
	Fit[a]	linear	linear	linear	quadratic	linear	linear
	Average posterior probability	0.941	0.934	0.958	0.954	0.943	0.982
	Odds of correct classification[b]	30.8	13.2	137.6	25.1	17.2	911.1
	Observed probability[c]	0.354	0.501	0.146	0.456	0.483	0.061
LDL-C	N (% of total)	249 (45.2)	255 (46.3)	47 (8.5)	286 (41.0)	340 (48.7)	72 (10.3)
	Fit[a]	linear	linear	linear	linear	linear	linear
	Average posterior probability	0.921	0.909	0.918	0.938	0.944	0.951
	Odds of correct classification[b]	14.1	11.6	120.1	21.9	17.9	168.9
	Observed probability[c]	0.446	0.463	0.091	0.403	0.491	0.105

**ln(Triglycerides)**	**N (% of total)**	178 (32.3)	279 (50.6)	94 (17.1)	258 (37.0)	319 (45.7)	121 (17.3)
	**Fit**[a]	linear	linear	linear	intercept	intercept	linear
	**Average posterior probability**	0.964	0.960	0.956	0.965	0.966	0.949
	**Odds of correct classification**[b]	56.18	23.12	104.47	46.5	33.9	89.5
	**Observed probability**[c]	0.328	0.498	0.173	0.367	0.464	0.169

[a]Modeled using a censored normal probability distribution for the dependent variable and blood-cholesterol lowering medication use as a time-dependent covariate in the decade prior to symptom onset, in cases, or matching age, in controls

[b]Based on the weighted posterior probability

[c]Group probability based on the posterior probabilities

Table S5. Chi-square tests of association of MCI and AD with lipid trajectory groups.

Lipid	Trajectory Group	MCI Case/Control Group			AD Case/Control Group		
		Controls[a] N = 435	Cases[a] N = 116	*p*[b]	Controls[a] N = 483	Cases[a] N = 215	*p*[b]
**Total Cholesterol**	1	116 (26.7)	38 (32.8)	0.095	221 (45.8)	98 (45.6)	0.396
	2	233 (53.6)	49 (42.2)		206 (42.6)	99 (46.0)	
	3	86 (19.8)	29 (25.0)		56 (11.6)	18 (8.4)	
**HDL-C**	1	233 (53.6)	73 (62.9)	0.089	209 (43.3)	94 (43.7)	**0.001***
	2	155 (35.6)	37 (31.9)		185 (38.3)	103 (47.9)	
	3	47 (10.8)	6 (5.4)		89 (18.4)	18 (8.4)	
**Non-HDL-C**	1	150 (34.5)	39 (33.6)	0.234	229 (47.4)	86 (40.0)	0.167
	2	229 (52.6)	55 (47.4)		226 (40.0)	117 (54.4)	
	3	56 (12.9)	22 (19.0)		28 (5.8)	12 (5.6)	
**LDL-C**	1	198 (45.5)	51 (44.0)	0.955	204 (42.2)	82 (38.1)	0.528
	2	200 (46.0)	55 (47.4)		232 (48.0)	108 (50.2)	
	3	37 (8.5)	10 (8.6)		47 (9.7)	25 (11.6)	
**ln(Triglycerides)**	1	150 (34.5)	28 (24.1)	0.050	178 (36.8)	80 (37.2)	0.632
	2	209 (48.0)	70 (60.3)		217 (44.9)	102 (47.4)	
	3	76 (17.5)	18 (15.5)		88 (18.2)	33 (15.4)	

[a]Number (percent of the total number of cases or controls)

[b]χ^2 test. An asterisk identifies *p* values remaining significant after Bonferroni adjustment.

Table S6. Cohort characteristics by quintile of total-cholesterol variability independent of the mean (VIM).

AD Case/Control Cohort (215 cases, 483 controls)							
**Characteristics**[a]		**VIM Quintile**[b]					***p***[c]
		**1**	**2**	**3**	**4**	**5**	
**Number of cases/controls**	**controls**	78 (16.1)	102 (21.1)	108 (22.4)	97 (20.1)	98 (20.3)	**0.002***
	**cases**	62 (28.8)	37 (17.2)	32 (14.9)	42 (19.5)	42 (19.5)	
	**total**	140 (20.1)	139 (19.9)	140 (20.1)	139 (19.9)	140 (20.1)	–
**Age, md (r)**		77 (51-92)	77 (52-93)	78 (51-91)	77 (52-94)	78 (52-93)	0.746
**Body Mass Index (BMI), md (r)**		26 (16-38)	25 (17-38)	26 (18-39)	25 (17-40)	26 (16-39)	0.624
**Female**		68 (19.2)	74 (20.8)	73 (20.6)	72 (20.3)	68 (19.2)	0.903
**Race**	**Caucasian**	135 (20.9)	126 (19.5)	128 (19.8)	128 (19.8)	128 (19.8)	0.561
	**Black/African American**	3 (15)	6 (30)	5 (25)	2 (10)	4 (20)	
	**Asian**	2 (7)	6 (21)	5 (17)	8 (28)	8 (28)	
	**Other**	0 (0)	1 (25)	2 (50)	1 (25)	0 (0)	
**Blood-cholesterol lowering medications used**		70 (13.4)	88 (16.8)	103 (19.7)	125 (23.9)	137 (26.2)	**< 0.001***
***APOE* ε2 Alleles**	**0**	125 (20.3)	117 (19.0)	119 (19.3)	126 (20.4)	130 (21.1)	0.180
	**1**	15 (19.5)	20 (26.0)	19 (24.7)	13 (16.9)	10 (13.0)	
	**2**	0 (0)	2 (50)	2 (50)	0 (0)	0 (0)	
***APOE* ε4 Alleles**	**0**	85 (19.8)	87 (20.3)	87 (20.3)	81 (18.9)	89 (20.8)	0.794
	**1**	44 (19.6)	43 (19.1)	48 (21.3)	50 (22.2)	40 (17.8)	
	**2**	11 (25.0)	9 (20.4)	5 (11.4)	8 (18.2)	11 (25.0)	
**Years of education**	**<12**	2 (13)	5 (33)	1 (7)	5 (33)	2 (13)	0.342

	**12**	19 (18.1)	26 (24.7)	22 (21.0)	16 (15.2)	22 (21.0)	
	**12-15**	15 (13.3)	26 (23.0)	23 (20.3)	25 (22.1)	24 (21.2)	
	**≥16**	104 (22.4)	82 (17.6)	94 (20.2)	93 (20.0)	92 (19.8)	
**Smoking**		1 (8)	4 (33)	3 (25)	1 (8)	3 (25	0.548
**Moderate-to-heavy alcohol use**		17 (23.3)	13 (17.8)	13 (17.8)	13 (17.8)	17 (23.3)	0.841
**Hypertension**		92 (17.7)	110 (21.2)	105 (20.2)	107 (20.6)	106 (20.4)	0.098
**Atherosclerosis**		5 (11)	6 (13)	9 (20)	13 (29)	12 (27)	0.202
**Cerebrovascular disease**		21 (15.1)	31 (22.3)	29 (20.9)	24 (17.3)	34 (24.5)	0.291
**Diabetes**		21 (11.7)	29 (16.1)	41 (22.8)	49 (27.2)	40 (22.2)	**0.001***
**Ischemic heart disease or myocardial infarction**		22 (9.9)	43 (19.3)	45 (20.2)	46 (20.6)	67 (30.0)	**< 0.001***
**Malignant neoplasm**		51 (18.2)	62 (22.1)	60 (21.4)	49 (17.5)	58 (20.7)	0.425
**Total Cholesterol Trajectory Group**	**1**	78 (24.4)	64 (20.1)	68 (21.3)	61 (19.1)	48 (15.0)	**0.044**
	**2**	51 (16.7)	64 (21.0)	55 (18.0)	61 (20.0)	74 (24.3)	
	**3**	11 (14.9)	11 (14.9)	17 (23.0)	17 (23.0)	18 (24.3)	
**HDL-C Trajectory Group**	**1**	57 (18.8)	57 (18.8)	62 (20.5)	58 (19.1)	69 (22.8)	**0.030**
	**2**	50 (17.4)	58 (20.1)	58 (20.1)	60 (20.8)	62 (21.5)	
	**3**	13 (30.8)	24 (22.4)	20 (18.7)	21 (19.6)	9 (8.4)	
**non-HDL-C Trajectory Group**	**1**	76 (24.1)	67 (21.3)	71 (22.5)	56 (17.8)	45 (14.3)	**0.005**
	**2**	59 (17.2)	66 (19.2)	64 (18.7)	73 (21.3)	81 (23.6)	
	**3**	5 (12)	6 (15)	5 (12)	10 (25)	14 (35)	
**LDL-C Trajectory Group**	**1**	64 (22.4)	62 (21.7)	66 (23.1)	54 (18.9)	40 (14.0)	**0.009**
	**2**	66 (19.4)	68 (20.0)	61 (17.9)	63 (18.5)	82 (24.1)	
	**3**	10 (13.9)	9 (12.5)	13 (18.0)	22 (30.6)	18 (25.0)	

**ln(Triglycerides) Trajectory Group**	**1**	69 (26.7)	46 (17.8)	45 (17.4)	46 (17.8)	52 (20.2)	0.112
	**2**	54 (16.9)	68 (21.3)	67 (21.0)	66 (20.7)	64 (20.1)	
	**3**	17 (14.0)	25 (20.7)	28 (23.1)	27 (22.3)	24 (19.8)	
**MCI Case/Control Cohort (116 cases, 435 controls)**							
**Characteristics**[a]		**VIM Quintile**[b]					***p***[c]
		**1**	**2**	**3**	**4**	**5**	
**Number of cases/controls**	**controls**	76 (17.5)	89 (20.5)	85 (19.5)	89 (20.5)	96 (22.1)	**0.027**
	**cases**	34 (29.3)	21 (18.1)	25 (21.6)	21 (18.1)	15 (12.9)	
	**total**	110 (20.0)	110 (20.0)	110 (20.0)	110 (20.0)	111 (20.2)	–
**Age, md (r)**		76 (53-98)	75 (51-92)	77 (51-93)	77 (52-93)	76 (55-94)	0.544
**BMI, md (r)**		26 (17-38)	26 (17-45)	27 (16-44)	27 (16-52)	27 (19-41)	0.207
**Female**		49 (18.8)	44 (16.9)	56 (21.5)	55 (21.2)	56 (21.5)	0.403
**Race**	**Caucasian**	103 (20.4)	103 (20.4)	100 (19.8)	102 (20.2)	96 (19.0)	0.158
	**Black/African American**	2 (17)	2 (17)	3 (25)	0 (0)	5 (42)	
	**Asian**	5 (17.2)	3 (10.3)	4 (13.8)	7 (24.1)	10 (34.5)	
	**Other**	0 (0)	2 (33)	3 (50)	1 (17)	0 (0)	
**Blood-cholesterol lowering medications used**		61 (14.0)	80 (18.3)	86 (19.7)	102 (23.3)	108 (24.7)	**< 0.001***
***APOE* ε2 Alleles**	**0**	95 (19.8)	95 (19.8)	93 (19.3)	104 (21.6)	94 (19.5)	0.212
	**1**	15 (22.1)	15 (22.1)	17 (25.0)	5 (7.4)	16 (23.5)	
	**2**	0 (0)	0 (0)	0 (0)	1 (50)	1 (50)	
***APOE* ε4 Alleles**	**0**	83 (21.3)	76 (19.5)	75 (19.3)	77 (19.8)	78 (20.0)	0.472
	**1**	25 (17.0)	32 (21.8)	34 (23.1)	29 (19.7)	27 (18.4)	

	**2**	2 (13)	2 (13)	1 (7)	4 (27)	6 (40)	
**Years of education**	**<12**	3 (25)	1 (8)	1 (8)	5 (42)	2 (17)	0.215
	**12**	14 (18.0)	13 (16.7)	14 (18.0)	20 (25.6)	17 (21.8)	
	**12-15**	15 (14.3)	19 (18.1)	20 (19.0)	28 (26.7)	23 (21.9)	
	**≥16**	78 (21.9)	77 (21.6)	75 (21.1)	57 (16.0)	69 (19.4)	
**Smoking**		3 (25)	1 (8	4 (33)	1 (8)	3 (25)	0.548
**Moderate-to-heavy alcohol use**		9 (14.3)	13 (20.6)	13 (20.6)	15 (23.8)	13 (20.6)	0.788
**Hypertension**		75 (17.6)	79 (18.5)	84 (19.7)	91 (21.3)	98 (22.9)	**0.002***
**Atherosclerosis**		6 (16.2)	4 (10.8)	4 (10.8)	11 (29.7)	12 (32.4)	0.076
**Cerebrovascular disease**		20 (21.3)	20 (21.3)	16 (17.0)	20 (21.3)	18 (19.2)	0.933
**Diabetes**		21 (12.9)	26 (16.0)	36 (22.2)	38 (23.5)	41 (25.3)	**0.014**
**Ischemic heart disease or myocardial infarction**		27 (13/3)	42 (20.7)	41 (20.2)	45 (2.2)	48 (23.6)	**0.041**
**Malignant neoplasm**		49 (21.0)	43 (18.5)	48 (20.6)	51 (21.9)	42 (18.0)	0.659
**Total Cholesterol Trajectory Group**	**1**	29 (18.8)	39 *25.3)	31 (20.1)	31 (20.1)	24 (15.6)	0.307
	**2**	58 (20.6)	57 (20.2)	54 (19.2)	52 (18.4)	61 (21.6)	
	**3**	23 (20.0)	14 (12.2)	25 (21.7)	27 (23.5)	26 (22.6)	
**HDL-C Trajectory Group**	**1**	50 (16.3)	68 (22.2)	65 (21.2)	56 (18.3)	67 (21.9)	0.191
	**2**	44 (22.9)	36 (18.8)	36 (18.8)	42 (21.9)	34 (17.7)	
	**3**	16 (30.2)	6 (11.3)	9 (17.0)	12 (22.6)	10 (18.9)	
**non-HDL-C Trajectory Group**	**1**	43 (22.8)	47 (24.9)	39 (20.6)	33 (17.5)	27 (14.3)	0.088
	**2**	54 (19.0)	54 (19.0)	55 (19.4)	59 (20.8)	62 (21.8)	
	**3**	13 (16.7)	9 (11.5)	16 (20.5)	18 (23.1)	22 (28.2)	
	**1**	52 (20.9)	55 (2.1)	54 (21.7)	49 (19.7)	39 (15.7)	0.283

**LDL-C Trajectory Group**	**2**	52 (20.4)	48 (18.8)	47 (18.4)	51 (20.0)	57 (22.4)	
	**3**	6 (12.8)	7 (14.9)	9 (19.2)	10 (21.3)	15 (31.9)	
**ln(Triglycerides) Trajectory Group**	**1**	43 (24.2)	39 (21.9)	34 (19.1)	23 (19.1)	38 (15.7)	0.132
	**2**	55 (19.7)	58 (20.8)	52 (18.6)	57 (20.4)	57 (20.4)	
	**3**	12 (12.8)	13 (13.8)	24 (25.5)	19 (20.2)	26 (27.7)	

[a]Age, BMI and social and medical history information are reported for age at first symptom onset, in cases, or matching age, in controls.

[b]VIM quintile and total-cholesterol trajectory group were determined as described in the text using total-cholesterol levels obtained over the 11-year period prior to and including the year of onset, in cases, or matching age, in controls. Number (percent of row total); for age and body mass index (BMI), median (md) [range(r)].

[c]Kruskal-Wallis test for age and BMI, otherwise χ^2 test; values <0.05 are in bold. An asterisk identifies *p* values remaining significant after Bonferroni adjustment.

Table S7. Cohort characteristics by quintile of non-HDL-C variability independent of the mean (VIM).

AD Case/Control Cohort (215 cases, 483 controls)							
**Characteristics**[a]		**VIM Quintile**[b]					***p***[c]
		**1**	**2**	**3**	**4**	**5**	
**Number of cases/controls**	**controls**	81 (16.8)	99 (20.5)	105 (21.7)	99 (20.5)	99 (20.5)	**0.023**
	**cases**	59 (27.4)	40 (18.6)	35 (16.3)	40 (18.5)	41 (19.1)	
	**total**	140 (20.1)	139 (19.9)	140 (20.1)	139 (19.9)	140 (20.1)	–
**Age, md (r)**		76 (51-92)	77 (52-93)	78 (52-92)	78 (52-94)	78 (52-93)	0.215
**Body Mass Index (BMI), md (r)**		26 (16-38)	25 (16-39)	26 (18-39)	25 (17-40)	26 (16-39)	0.700
**Female**		76 (21.4)	72 (20.3)	65 (18.3)	67 (18.9)	75 (21.1)	0.625
**Race**	**Caucasian**	130 (20.2)	129 (20.0)	130 (20.2)	128 (19.8)	128 (19.8)	0.374
	**Black/African American**	8 (40)	3 (15)	2 (10)	3 (15)	4 (20)	
	**Asian**	2 (7)	5 (17)	7 (24)	7 (24)	8 (28)	
	**Other**	0 (0)	2 (50)	1 (25)	1 (25)	0 (0)	
**Blood-cholesterol lowering medications used**		67 (12.8)	87 (16.6)	114 (21.8)	120 (22.9)	135 (25.8)	**< 0.001***
***APOE* ε2 Alleles**	**0**	129 (20.9)	119 (19.3)	117 (19.0)	122 (19.8)	150 (21.1)	0.223
	**1**	11 (14.3)	19 (24.7)	21 (27.3)	16 (20.8)	10 (13.0)	
	**2**	0 (0)	1 (25)	2 (50)	1 (25)	0 (0)	
***APOE* ε4 Alleles**	**0**	81 (18.9)	91 (21.2)	85 (19.8)	83 (19.4)	89 (20.8)	0.457
	**1**	44 (19.6)	42 (18.7)	48 (21.3)	49 (21.8)	42 (18.7)	
	**2**	15 (34.1)	6 913.6)	7 (15.9)	7 (15.9)	9 (20.4)	
**Years of education**	**<12**	4 (27)	2 (13)	3 (20)	2 (13)	4 (27)	0.837

	**12**	24 (22.9)	22 (21.0)	17 (16.2)	19 (18.1)	23 (21.9)	
	**12-15**	17 (15.0)	19 (16.8)	28 (24.8)	27 (23.9)	22 (19.5)	
	**≥16**	95 (20.4)	96 (20.6)	92 (19.8)	91 (19.6)	91 (19.6)	
**Smoking**		3 (25)	2 (17)	4 (33)	0 (0)	3 (25)	0.424
**Moderate-to-heavy alcohol use**		16 (21.9)	10 (13.7)	15 (20.6)	16 (21.9)	16 (21.9)	0.729
**Hypertension**		98 (18.8)	91 (17.5)	114 (21.9)	108 (20.8)	109 (21.0)	**0.014**
**Atherosclerosis**		6 (13.3)	5 (11.1)	9 (20.0)	13 (28.9)	12 (26.7)	0.203
**Cerebrovascular disease**		24 (17.3)	27 (19.4)	33 (23.7)	22 (15.8)	33 (23.7)	0.343
**Diabetes**		19 (10.6)	27 (15.0)	48 (26.7)	47 (26.1)	39 (21.7)	**< 0.001***
**Ischemic heart disease or myocardial infarction**		22 (9.9)	33 (14.8_	55 (24.7)	43 (19.3)	70 (31.4)	**< 0.001***
**Malignant neoplasm**		60 (21.4)	54 (19.3)	59 (21.1)	50 (17.9)	57 (20.4)	0.775
**Total Cholesterol Trajectory Group**	**1**	68 (21.3)	67 (21.0)	74 (23.2)	65 (20.4)	45 (14.1)	**0.029**
	**2**	58 (19.0)	61 (20.0)	49 (16.1)	58 (19.0)	79 (25.9)	
	**3**	14 (18.9)	11 (14.9)	17 (23.0)	16 (21.6)	16 (21.6)	
**HDL-C Trajectory Group**	**1**	58 (19.1)	58 (19.1)	65 (21.4)	59 (19.5)	63 (20.8)	0.543
	**2**	53 (18.4)	60 (20.8)	52 (18.1)	62 (21.5)	61 (21.2)	
	**3**	29 (27.1)	21 (19.6)	23 (21.5)	18 (16.8)	16 (15.0)	
**non-HDL-C Trajectory Group**	**1**	67 (21.3)	66 (21.0)	71 (22.5)	58 (18.4)	54 (16.8)	0.378
	**2**	67 (19.5)	67 (19.5)	63 (18.4)	71 (20.7)	75 (21.9)	
	**3**	6 (15.0)	6 915.0)	6 (15.0)	10 (25.0)	12 (30.0)	
**LDL-C Trajectory Group**	**1**	58 (20.3)	59 (20.6)	70 (24.5)	54 (18.9)	45 (15.7)	0.071
	**2**	71 (20.9)	67 (19.7)	57 (16.8)	64 (18.8)	81 (23.8)	
	**3**	11 (15.3)	13 (18.1)	13 (18.1)	21 (29.2)	14 (19.4)	

**ln(Triglycerides) Trajectory Group**	**1**	57 (22.1)	55 (21.3)	49 (19.0)	41 (15.9)	56 (21.7)	0.657
	**2**	61 (19.1)	59 (18.5)	65 (20.4)	71 (22.3)	63 (19.8)	
	**3**	22 (18.2)	25 (20.7)	26 (21.5)	27 (22.3)	21 (17.4)	
**MCI Case/Control Cohort (116 cases, 435 controls)**							
**Characteristics**[a]		**VIM Quintile**[b]					***p***[c]
		**1**	**2**	**3**	**4**	**5**	
**Number of cases/controls**	**controls**	74 (17.0)	85 (19.5)	93 (21.4)	89 (20.5)	94 (21.6)	**0.008**
	**cases**	36 (31.0)	25 (21.6)	17 (14.7)	21 (18.1)	17 (14.7)	
	**total**	110 (20.0)	110 (20.0)	110 (20.0)	110 (20.0)	111 (20.2)	–
**Age, md (r)**		76 (51-91)	75 (51-98)	76 (52-89)	78 (53-94)	76 (54-94)	**0.019**
**BMI, md (r)**		26 (17-40)	26 (17-46)	27 (19-52)	26 (16-43)	27 (16-41)	0.462
**Female**		50 (19.2)	53 920.4)	42 (16.20	57 (21.9)	58 (22.3)	0.211
**Race**	**Caucasian**	105 920.8)	103 (20.4)	98 (19.4)	101 (20.0)	97 (19.2)	**0.028**
	**Black/African American**	2 (17)	0 (0)	5 942)	1 (8)	4 (33)	
	**Asian**	3 (10)	4 (14)	4 (14)	8 (28)	10 (34)	
	**Other**	0 (0)	3 (50)	3 (50)	0 (0)	0 (0)	
**Blood-cholesterol lowering medications used**		63 (14.4)	76 (17.4)	90 (20.6)	98 (22.4)	110 (25.2)	**< 0.001***
***APOE* ε2 Alleles**	**0**	99 (20.6)	98 (20.4)	92 (19.1)	96 (20.0)	96 (20.0)	0.773
	**1**	11 (16.2)	12 (17.6)	17 (25.0)	14 (20.6)	14 (20.6)	
	**2**	0 (0)	0 (0)	1 (50)	0 (0)	1 (50)	
***APOE* ε4 Alleles**	**0**	73 (18.8)	92 (21.1)	80 (20.6)	76 (19.5)	78 (20.0)	0.756
	**1**	34 (23.1)	27 (18.4)	28 (19.0)	30 (20.4)	28 (19.0)	
	**2**	3 (20)	1 (7)	2 (13)	4 (27)	5 (33)	

**Years of education**	**<12**	3 (25)	0 (0)	4 (33)	2 (17)	3 (25)	0.654
	**12**	15 (19.2)	14 (18.0)	13 (16.7)	17 (21.8)	19 (24.4)	
	**12-15**	16 (15.2)	19 (18.1)	22 (21.0)	26 (24.8)	22 (21.0)	
	**≥16**	76 (21.4)	77 (21.6)	71 (19.9)	65 (18.3)	67 (18.8)	
**Smoking**		3 (25)	1 (8)	3 (25)	2 (17)	3 (25)	0.852
**Moderate-to-heavy alcohol use**		10 (15.9)	11 (17.5)	14 (22.2)	12 (19.0)	16 (25.4)	0.735
**Hypertension**		73 (17.1)	75 (17.6)	92 (21.6)	88 (20.6)	99 (23.2)	**< 0.001***
**Atherosclerosis**		7 (19)	4 (11)	4 (11)	8 (22)	14 (38)	**0.048**
**Cerebrovascular disease**		18 (19.2)	17 (18.1)	15 (16.0)	25 (26.6)	19 (20.2)	0.456
**Diabetes**		20 (12.4)	25 (15.3)	39 (24.1)	39 (24.1)	29 (24.1)	**0.005**
**Ischemic heart disease or myocardial infarction**		28 (13.8)	36 (17.7)	45 (22.2)	45 (22.2)	49 (24.1)	**0.026**
**Malignant neoplasm**		49 (21.0)	46 (19.7	49 (21.0)	47 (20.2)	42 (18.0)	0.848
**Total Cholesterol Trajectory Group**	**1**	31 (20.1)	31 (20.1)	36 (23.4)	26 (16.9)	30 (19.5)	0.803
	**2**	59 (20.9)	59 (20.9)	49 (17.4)	57 (20.2)	58 (20.6)	
	**3**	20 (17.4)	20 (17.4)	25 (21.7)	27 (23.5)	23 (20.0)	
**HDL-C Trajectory Group**	**1**	54 (17.7)	68 (22.2)	65 (21.2)	56 (18.3)	63 (20.6)	0.262
	**2**	44 (22.9)	35 (18.2)	38 (19.8)	37 (19.3)	38 (19.8)	
	**3**	12 (22.6)	7 (13.2)	7 (13.2)	17 (32.1)	10 (18.9)	
**non-HDL-C Trajectory Group**	**1**	43 (22.8)	24 (18.5)	43 (22.8)	31 (16.4)	37 (19.6)	0.457
	**2**	54 (19.0)	61 (21.5)	48 (16.9)	65 (22.9)	56 (19.7)	
	**3**	13 (16.7)	14 (18.0)	19 (24.4)	14 (18.0)	18 (23.1)	
**LDL-C Trajectory Group**	**1**	49 (19.7)	47 (18.9)	58 (23.3)	47 (18.9)	48 (19.3)	0.634
	**2**	55 921.6)	53 (20.8)	41 (16.1)	54 (21.2)	52 (20.4)	

	**3**	6 (12.8)	10 (21.3)	11 (23.4)	9 (19.2)	11 (23.4)	
**ln(Triglycerides) Trajectory Group**	**1**	40 (22.5)	35 (19.7)	35 (19.7)	35 (19.7)	33 (18.5)	0.632
	**2**	59 (21.2)	57 (20.4)	55 (19.7)	54 (19.4)	54 (19.4)	
	**3**	11 (11.7)	18 (19.2)	20 (21.3)	21 (22.3)	24 (25.5)	

[a]Age, BMI and social and medical history information are reported for age at first symptom onset, in cases, or matching age, in controls.

[b]VIM quintile and total-cholesterol trajectory group were determined as described in the text using total-cholesterol levels obtained over the 11-year period prior to and including the year of onset, in cases, or matching age, in controls. Number (percent of row total); for age and body mass index (BMI), median (md) [range(r)].

[c]Kruskal-Wallis test for age and BMI, otherwise χ^2 test; values <0.05 are in bold. An asterisk identifies *p* values remaining significant after Bonferroni adjustment.

Table S8. Cohort characteristics by quintile of HDL-C variability independent of the mean (VIM).

AD Case/Control Cohort (215 cases, 483 controls)							
**Characteristics**[a]		**VIM Quintile**[b]					***p***[c]
		**1**	**2**	**3**	**4**	**5**	
**Number of cases/controls**	**controls**	95 (19.7)	98 (20.3)	95 (19.7)	95 (19.7)	100 (20.7)	0.949
	**cases**	45 (20.9)	41 (19.1)	45 (20.9)	44 (20.5)	40 (18.6)	
	**total**	140 (20.1)	139 (19.9)	140 (20.1)	139 (19.9)	140 (20.1)	–
**Age, md (r)**		79 (52-90)	76 (52-93)	78 (51-94)	77 (52-89)	77 (51-93)	0.555
**Body Mass Index (BMI), md (r)**		26 (17-36)	26 (16-39)	25 (16-40)	25 (18-39)	26 (18-37)	0.373
**Female**		76 (21.4)	62 (17.5)	73 (20.6)	72 (20.3)	72 (20.3)	0.559
**Race**	**Caucasian**	132 (20.5)	127 (19.7)	128 (19.8)	128 (19.8)	130 (20.2)	**0.032**
	**Black/African American**	2 (10)	2 (10)	3 915)	5 (25)	8 (40)	
	**Asian**	6 (21)	9 (31)	9 (31)	3 (10)	2 (7)	
	**Other**	0 (0)	1 (25)	0 (0)	3 (75)	0 (0)	
**Blood-cholesterol lowering medications used**		104 (19.9)	104 (19.9)	102 (19.5)	108 (20.6)	105 (20.1)	0.922
***APOE* ε2 Alleles**	**0**	127 (20.6)	124 (20.1)	119 (19.3)	123 (19.9)	124 (20.1)	0.153
	**1**	13 (16.9)	12 (15.6)	21 (27.3)	15 (19.5)	16 (20.8)	
	**2**	0 (0)	3 (75)	0 (0)	1 (25)	0 (0)	
***APOE* ε4 Alleles**	**0**	86 (20.0)	88 (20.5)	88 (20.5)	85 (19.8)	82 (19.1)	0.477
	**1**	49 (21.8)	46 (20.4)	39 (17.3)	44 (19.6)	47 (20.9)	
	**2**	5 (11.4)	5 (11.4)	13 (29.6)	10 (22.7)	11 (25.0)	
**Years of education**	**<12**	3 (20)	4 (27)	3 (20)	2 (13)	3 (20)	0.855

	**12**	23 (21.9)	19 (18.1)	21 (20.0)	26 (24.8)	16 (15.2)	
	**12-15**	23 (20.4)	28 (24.8)	19 (16.8)	23 (20.4)	20 (17.7)	
	**≥16**	91 (19.6)	88 (18.9)	97 (20.9)	88 (18.9)	101 (21.7)	
**Smoking**		3 (25)	3 (25)	2 (17)	2 (17)	2 (17)	0.972
**Moderate-to-heavy alcohol use**		10 (13.7)	9 (12.3)	18 (24.7)	11 (15.1)	25 (24.3)	**0.007**
**Hypertension**		106 (20.4)	107 (20.6)	104 (20.0)	94 (18.1)	109 (21.0)	0.303
**Atherosclerosis**		4 (8.9)	9 (20.0)	7 (15.6)	11 (24.4)	14 (31.1)	0.141
**Cerebrovascular disease**		26 (18.7)	29 (20.9)	32 (23.0)	25 (18.0)	27 (19.4)	0.852
**Diabetes**		35 (19.4)	42 (23.3)	31 (17.2)	34 (18.9)	38 (21.1)	0.609
**Ischemic heart disease or myocardial infarction**		37 (16.6)	43 (19.3)	43 (19.3)	47 (21.1)	53 (23.8)	0.331
**Malignant neoplasm**		49 (17.5)	53 (18.9)	62 (22.1)	61 (21.8)	55 (19.6)	0.459
**Total Cholesterol Trajectory Group**	**1**	62 919.4)	57 (17.9)	64 (20.1)	62 (19.4)	74 (23.2)	0.431
	**2**	64 (21.0)	70 (23.0)	62 (20.3)	57 (18.7)	52 (17.0)	
	**3**	14 (18.9)	12 (16.2)	14 (18.9)	20 (27.0)	14 (18.9)	
**HDL-C Trajectory Group**	**1**	65 (21.4)	68 (22.4)	53 (17.5)	64 (21.1)	53 (17.5)	0.067
	**2**	50 (17.4)	48 (16.7)	60 (20.8)	58 (20.1)	72 (25.0)	
	**3**	25 (23.4)	23 (21.5)	27 (25.2)	17 (15.9)	15 (14.0)	
**non-HDL-C Trajectory Group**	**1**	57 (18.1)	67 (21.3)	63 (20.0)	59 (18.7)	69 (21.9)	0.346
	**2**	75 (21.9)	63 (18.4)	73 (21.3)	67 (19.5)	65 (19.0)	
	**3**	8 (20.0)	9 (22.4)	4 (10.0)	13 (32.5)	6 (15.0)	
**LDL-C Trajectory Group**	**1**	56 (19.6)	50 (17.5)	57 (19.9)	57 (19.9)	66 (23.1)	0.427
	**2**	72 (21.2)	73 (21.5)	72 (21.2)	62 (18.2)	61 (17.9)	
	**3**	12 (16.7)	16 (22.2)	11 (15.3)	20 (27.8)	13 (18.1)	

**ln(Triglycerides) Trajectory Group**	**1**	54 (20.9)	54 (20.9)	49 (19.0)	50 (19.4)	51 (19.8)	0.882
	**2**	58 (18.2)	59 (18.5)	71 (22.3)	65 (20.4)	66 (20.7)	
	**3**	28 (23.1)	26 (21.5)	20 (16.5)	24 (19.8)	23 (19.1)	
**MCI Case/Control Cohort (116 cases, 435 controls)**							
**Characteristics**[a]		**VIM Quintile**[b]					***p***[c]
		**1**	**2**	**3**	**4**	**5**	
**Number of cases/controls**	**controls**	86 (19.8)	91 (20.9)	84 (19.3)	86 (19.8)	88 (20.2)	0.832
	**cases**	24 (20.7)	19 (16.4)	26 (22.4)	24 (20.7)	23 (19.8)	
	**total**	110 (20.0)	110 (20.0)	110 (20.0)	110 (20.0)	111 (20.2)	–
**Age, md (r)**		75 (51-98)	76 (53-93)	75 (53-92)	76 (55-94)	77 (51-94)	0.680
**BMI, md (r)**		27 (18-41)	27 (16-43)	26 (17-40)	27 (18-52)	26 (16-41)	0.571
**Female**		45 (17.3)	50 (19.2)	52 (20.0)	56 (21.5)	57 (21.9)	0.516
**Race**	**Caucasian**	103 (20.4)	97 (19.2)	103 (20.4)	100 (19.8)	101 (20.0)	0.808
	**Black/African American**	2 (17)	2 (17)	1 (8)	3 (25)	4 (33)	
	**Asian**	3 (10)	10 (35)	5 (17)	6 (21)	5 (17)	
	**Other**	2 (33)	1 (17)	1 (17)	1 (17)	1 (17)	
**Blood-cholesterol lowering medications used**		85 (19.4)	85 (19.4)	87 (19.9)	92 (21.0)	88 (20.1)	0.770
***APOE* ε2 Alleles**	**0**	96 (20.0)	99 (20.6)	98 (20.4)	92 (19.1)	96 (20.0)	0.643
	**1**	13 (19.1)	10 (14.7)	12 (17.6)	18 (26.5)	15 (22.1)	
	**2**	1 (50)	1 (50)	0 (0)	0 (0)	0 (0)	
***APOE* ε4 Alleles**	**0**	83 (21.3)	71 (18.2)	78 (20.0)	83 (21.3)	74 (19.0)	**0.022**
	**1**	25 (17.0)	39 (26.5)	29 (19.7)	25 (17.0)	29 (19.7)	
	**2**	2 (13)	0 (0)	3 (20)	2 (13)	8 (53)	

**Years of education**	**<12**	3 (25)	2 (17)	3 (25)	3 (25)	1 (8)	0.693
	**12**	15 (19.2)	15 (19.2)	15 (19.2)	10 (12.8)	23 (29.5)	
	**12-15**	17 (16.2)	22 (21.0)	20 (19.0)	25 (23.8)	21 (20.0)	
	**≥16**	75 (21.1)	71 (19.9)	72 (20.2)	72 (20.2)	66 (18.5)	
**Smoking**		3 (25)	2 (17)	3 (25)	3 (25)	1 (7)	0.847
**Moderate-to-heavy alcohol use**		11 (17.5)	8 (12.7)	8 (12.7)	17 (27.0)	19 (30.2)	0.054
**Hypertension**		79 (18.5)	90 (21.1)	79 (18.5)	89 (20.8)	90 (21.1)	0.147
**Atherosclerosis**		3 (8.1)	4 (10.8)	7 (18.9)	12 (32.4)	11 (29.7)	0.052
**Cerebrovascular disease**		16 (17.0)	20 (21.3)	16 917.0)	27 (28.7)	15 (15.9)	0.170
**Diabetes**		28 (17.3)	38 (23.5)	29 (17.9)	41 (25.3)	26 (16.0)	0.095
**Ischemic heart disease or myocardial infarction**		34 (16.8)	34 (16.8)	42 (20.7)	52 (25.6)	41 (20.2)	0.073
**Malignant neoplasm**		49 (21.0)	46 (19.7)	42 (18.0)	48 (20.6)	48 (20.6)	0.891
**Total Cholesterol Trajectory Group**	**1**	28 (18.2)	26 (16.9)	27 (17.5)	37 (24.0)	36 (23.4)	0.643
	**2**	59 (20.9)	56 (19.9)	59 (20.9)	54 (19.2)	54 (19.2)	
	**3**	23 (20.0)	28 (24.4)	24 (20.9)	19 (16.5)	21 (18.3)	
**HDL-C Trajectory Group**	**1**	70 (22.9)	64 (20.9)	59 (19.3)	62 (20.3)	51 (16.7)	0.320
	**2**	31 (16.2)	34 (17.7)	39 (20.3)	38 (19.8)	50 (26.0)	
	**3**	9 (17.0)	12 (22.6)	12 (22.6)	10 (18.9)	10 (18.9)	
**non-HDL-C Trajectory Group**	**1**	34 (18.0)	34 (18.0)	32 (16.9)	43 (22.8)	46 (24.3)	0.520
	**2**	58 (20.4)	60 21.1)	64 (22.5)	51 (18.0)	51 (18.0)	
	**3**	18 (23.1)	16 (20.5)	14 (18.0)	16 (20.5)	14 (18.0)	
**LDL-C Trajectory Group**	**1**	44 (17.7)	49 (19.7)	46 (18.5)	56 (22.5)	54 (21.7)	0.636
	**2**	56 (22.0)	51 (20.0)	57 (22.4)	46 (18.0)	45 (17.7)	

	**3**	10 (21.3)	10 (21.3)	7 (14.9)	8 (17.0)	12 (25.5)	
**ln(Triglycerides) Trajectory Group**	**1**	36 (20.2)	37 (20.8)	35 (19.7)	34 (19.1)	36 (20.2)	0.663
	**2**	53 (19.0)	53 (19.0)	55 (19.7)	54 (19.4)	64 (22.9)	
	**3**	21 (22.3)	20 (21.3)	20 (21.3)	22 (23.4)	11 (11.7)	

[a]Age, BMI and social and medical history information are reported for age at first symptom onset, in cases, or matching age, in controls.

[b]VIM quintile and HDL trajectory group were determined as described in the text using HDL levels obtained over the 11-year period prior to and including the year of onset, in cases, or matching age, in controls. Number (percent of row total); for age and body mass index (BMI), median (md) [range(r)].

[c]Kruskal-Wallis test for age and BMI, otherwise χ^2 test; values <0.05 are in bold. An asterisk identifies *p* values remaining significant after Bonferroni adjustment.

Table S9. Cohort characteristics by quintile of LDL-C variability independent of the mean (VIM).

AD Case/Control Cohort (215 cases, 483 controls)							
**Characteristics**[a]		**VIM Quintile**[b]					***p***[c]
		**1**	**2**	**3**	**4**	**5**	
**Number of cases/controls**	**controls**	88 (18.2)	96 (19.9)	100 (20.7)	99 (20.5)	100 (20.7)	0.468
	**cases**	52 (24.2)	43 (20.0)	40 (18.6)	40 (18.6)	40 (18.6)	
	**total**	140 (20.1)	139 (19.9)	140 (20.1)	139 (19.9)	140 (20.1)	–
**Age, md (r)**		76 (52-92)	77 (51-93)	78 (52-92)	78 (52-89)	78 (52-94)	0.413
**Body Mass Index (BMI), md (r)**		26 (16-39)	25 (16-39)	26 (18-40)	25 (17-38)	26 (16-39)	0.579
**Female**		79 (22.2)	68 (19.2)	65 (18.3)	65 (18.3)	78 (22.0)	0.258
**Race**	**Caucasian**	129 (20.0)	131 (20.3)	129 (20.0)	128 (19.8)	129 (19.8)	0.159
	**Black/African American**	7 (35)	4 920)	1 (5)	3 (15)	5 (25)	
	**Asian**	3 (10)	4 914)	7 (24)	8 (28)	7 (24)	
	**Other**	1 (25)	0 (0)	3 (75)	0 (0)	0 (0)	
**Blood-cholesterol lowering medications used**		74 (14.2)	85 (16.2)	106 (20.3)	122 (23.3)	136 (26.0)	**< 0.001***
***APOE* ε2 Alleles**	**0**	124 (20.1)	117 (19.0)	122 (19.8)	127 (20.6)	127 (20.6)	0.509
	**1**	15 (19.5)	21 (27.3)	16 (20.8)	12 (15.6)	13 (16.9)	
	**2**	1 (25)	1 (25)	2 (50)	0 (0)	0 (0)	
***APOE* ε4 Alleles**	**0**	88 (20.5)	83 (19.4)	88 (20.5)	80 (18.6)	90 (21.0)	0.656
	**1**	39 (17.3)	49 (21.8)	45 (20.0)	51 (22.7)	41 (18.2)	
	**2**	13 (29.6)	7 (15.9)	7 (15.9)	8 (18.2)	9 (20.4)	
**Years of education**	**<12**	5 (33)	1 (7)	2 (13)	4 (27)	3 (20)	0.646

	**12**	20 (19.0)	23 (21.9)	22 (21.0)	15 (14.3)	25 (23.8)	
	**12-15**	16 (14.2)	25 (22.1)	22 (19.5)	26 (23.0)	24 (21.2)	
	**≥16**	99 (21.3)	90 (19.4)	94 (20.2)	94 (20.2)	88 (18.9)	
**Smoking**		3 (25)	2 (17)	4 (33)	1 (8)	2 (17)	0.703
**Moderate-to-heavy alcohol use**		13 (17.8)	12 (16.4)	14 (19.2)	14 (19.2)	20 (27.4)	0.567
**Hypertension**		95 (18.3)	99 (19.0)	108 (20.8)	110 (21.2)	108 (20.8)	0.156
**Atherosclerosis**		6 (13.3)	5 (11.1)	11 (24.4)	11 (24.4)	12 (26.7)	0.291
**Cerebrovascular disease**		24 (17.3)	31 (22.3)	33 (23.7)	23 (16.60	28 (20.1)	0.504
**Diabetes**		17 (9.4)	36 (20.0)	38 (21.1)	53 (29.4)	36 (20.0)	**< 0.001***
**Ischemic heart disease or myocardial infarction**		30 (13.4)	31 (13.9)	51 (22.9)	48 (21.5)	63 (28.2)	**< 0.001***
**Malignant neoplasm**		59 (21.1)	55 (19.6)	61 (21.8)	49 (17.5)	56 (20.0)	0.677
**Total Cholesterol Trajectory Group**	**1**	68 (21.3)	74 (23.2)	71 (22.3)	61 (19.1)	45 (14.1)	**0.025**
	**2**	57 (18.7)	55 (18.0)	52 (17.0)	63 (20.7)	78 (25.6)	
	**3**	15 (20.3)	10 (13.5)	17 (23.0)	15 (20.3)	17 (23.0)	
**HDL-C Trajectory Group**	**1**	58 (19.1)	57 (18.8)	61 (20.1)	68 (22.4)	59 (19.5)	0.414
	**2**	53 (18.4)	57 (19.8)	61 (21.2)	53 (18.4)	64 (22.2)	
	**3**	29 *27.1)	25 (23.4)	18 (16.8)	18 (16.8)	17 (15.9)	
**non-HDL-C Trajectory Group**	**1**	70 (22.2)	71 (22.5)	68 (21.6)	53 (16.8)	53 (16.8)	**0.021**
	**2**	64 (18.7)	65 (19.0)	63 (18.4)	79 (23.0)	72 (21.0)	
	**3**	6 (15.0)	3 (7.5)	9 (22.5)	7 (17.5)	15 (37.5)	
**LDL-C Trajectory Group**	**1**	59 (20.6)	64 (22.4)	67 (23.4)	54 (18.9)	42 (14.7)	**0.042**
	**2**	72 (21.2)	62 (18.2)	60 (17.6)	65 (19.1)	81 (23.8)	
	**3**	9 (12.5)	13 (18.1)	13 (18.1)	20 (27.8)	17 (23.6)	

**ln(Triglycerides) Trajectory Group**	**1**	57 (22.1)	61 (23.60	44 (17.0)	44 (17.0)	52 (20.2)	0.079
	**2**	59 (18.5)	62 (19.4)	72 (22.6)	60 (18.8)	66 (20.7)	
	**3**	24 (19.8)	16 (13.2)	24 (19.8)	35 (28.9)	22 (18.2)	
**MCI Case/Control Cohort (116 cases, 435 controls)**							
**Characteristics**[a]		**VIM Quintile**[b]					***p***[c]
		**1**	**2**	**3**	**4**	**5**	
**Number of cases/controls**	**controls**	76 (17.5)	87 (20.0)	89 (20.5)	87 (20.0)	96 (22.1)	**0.033**
	**cases**	34 (29.3)	23 (19.8)	21 (18.1)	23 (19.8)	15 (12.9)	
	**total**	110 (20.0)	110 (20.0)	110 (20.0)	110 (20.0)	111 (20.2)	–
**Age, md (r)**		75 (51-98)	76 (51-92)	77 (52-93)	77 (53-94)	76 (55-94)	0.276
**BMI, md (r)**		26 (17-40)	26 (18-46)	26 (17-52)	27 (16-44)	27 (16-42)	0.566
**Female**		47 (18.1)	45 (17.3)	52 (20.0)	54 (20.8)	62 (23.8)	0.190
**Race**	**Caucasian**	109 (21.6)	100 (19.7)	97 (19.2)	100 (19.7)	98 (19.4)	**0.001***
	**Black/African American**	1 (8)	1 (8)	4 (33)	1 (8)	5 (42)	
	**Asian**	0 (0)	8 (27.6)	4 (13.8)	9 (31.0)	8 (27.6)	
	**Other**	0 (0)	1 (17)	5 (83)	0 (0)	0 (0)	
**Blood-cholesterol lowering medications used**		64 (14.6)	82 (18.8)	81 (18.5)	100 (22.9)	110 (25.2)	**< 0.001***
***APOE* ε2 Alleles**	**0**	99 (20.6)	94 (19.5)	93 (19.3)	99 (20.6)	96 (20.0)	0.642
	**1**	11 (16.2)	16 (23.5)	17 (25.0)	10 (14.7)	14 (20.6)	
	**2**	0 (0)	0 (0)	0 (0)	1 (50)	1 (50)	
***APOE* ε4 Alleles**	**0**	79 (20.3)	80 (20.6)	76 (19.5)	76 (19.5)	78 (20.0)	0.926
	**1**	28 (19.0)	29 (19.7)	31 (21.1)	31 (21.1)	28 (19.0)	
	**2**	3 (20)	1 (7)	3 (20)	3 (20)	5 (33)	

**Years of education**	**<12**	1 (8)	1 (8)	3 (25)	3 (25)	4 (33)	0.272
	**12**	17 (21.8)	9 (11.5)	17 (21.8)	17 (21.8)	18 (23.1)	
	**12-15**	13 (12.4)	24 (22.9)	19 (18.1)	22 (21.0)	27 (25.7)	
	**≥16**	79 (22.2)	76 (21.4)	71 (19.9)	68 (19.1)	62 (17.4)	
**Smoking**		2 (17)	1 (8)	4 (33)	2 (17)	3 (25)	0.698
**Moderate-to-heavy alcohol use**		11 (17.5)	11 (17.5)	12 (19.0)	14 (22.2)	15 (23.8)	0.889
**Hypertension**		74 (17.3)	79 (18.5)	84 919.7)	91 (21.3)	99 (23.2)	**0.001***
**Atherosclerosis**		6 (16.2)	3 (8.1)	6 (16.2)	12 (32.4)	10 (27.0)	0.117
**Cerebrovascular disease**		18 (19.2)	18 (19.2)	13 (13.8)	26 (27.7)	19 (20.2)	0.233
**Diabetes**		20 (12.4)	27 (16.7)	35 (21.6)	42 (25.9)	38 (23.5)	**0.009**
**Ischemic heart disease or myocardial infarction**		33 (16.3)	40 (19.7)	34 (16.8)	50 (24.6)	46 (22.7)	0.078
**Malignant neoplasm**		52 (22.3)	43 (18.4)	47 (20.2)	45 (19.3)	46 (19.7)	0.789
**Total Cholesterol Trajectory Group**	**1**	41 (26.6)	32 (20.8)	30 (19.5)	30 (19.5)	21 (13.6)	0.091
	**2**	54 (19.2)	59 (20.9)	52 (18.4)	56 (19.9)	61 (21.6)	
	**3**	15 (13.0)	19 (16.5)	28 (24.4)	24 (20.9)	29 (25.2)	
**HDL-C Trajectory Group**	**1**	58 (19.0)	62 (20.3)	67 (21.9)	57 (19.6)	62 (20.3)	0.435
	**2**	40 (20.8)	40 (20.8)	38 (19.8)	37 (19.3)	38 (19.3)	
	**3**	12 (22.6)	8 (15.1)	5 (9.4)	16 (30.2)	12 (22.6)	
**non-HDL-C Trajectory Group**	**1**	50 (26.5)	42 (22.2)	33 (17.5)	36 (19.0)	38 (14.8)	**0.018**
	**2**	51 (18.0)	55 (19.4)	55 (19.4)	62 (21.8)	61 (21.5)	
	**3**	9 (11.5)	13 (16.7)	22 (28.2)	12 (15.4)	22 (28.2)	
**LDL-C Trajectory Group**	**1**	60 (24.1)	51 (20.5)	44 (17.7)	57 (22.9)	37 (14.9)	**0.020**
	**2**	46 (18.0)	51 (20.0)	51 (20.0)	45 (17.6)	62 (24.3)	

	**3**	4 (8.5)	8 (17.0)	15 (31.9)	8 (17.0)	12 (25.5)	
**ln(Triglycerides) Trajectory Group**	**1**	43 (24.2)	39 (21.9)	29 (16.3)	36 (20.2)	31 (17.4)	0.139
	**2**	57 (20.4)	53 (19.0)	61 (21.9)	55 (19.7)	53 (19.0)	
	**3**	10 (10.6)	18 (19.2)	20 (21.3)	19 (20.2)	27 (28.7)	

[a]Age, BMI and social and medical history information are reported for age at first symptom onset, in cases, or matching age, in controls.

[b]VIM quintile and LDL-C trajectory group were determined as described in the text using LDL-C levels obtained over the 11-year period prior to and including the year of onset, in cases, or matching age, in controls. Number (percent of row total); for age and body mass index (BMI), median (md) [range(r)].

[c]Kruskal-Wallis test for age and BMI, otherwise χ^2 test; values <0.05 are in bold. An asterisk identifies *p* values remaining significant after Bonferroni adjustment.

Table S10. Cohort characteristics by quintile of ln(triglycerides) variability independent of the mean (VIM).

AD Case/Control Cohort (215 cases, 483 controls)							
**Characteristics**[a]		**VIM Quintile**[b]					***p***[c]
		**1**	**2**	**3**	**4**	**5**	
**Number of cases/controls**	**controls**	90 (18.6)	103 (21.3)	100 (20.7)	95 (19.7)	95 (19.7)	0.456
	**cases**	50 (23.3)	36 (16.7)	40 (18.6)	44 (20.5)	45 (20.9)	
	**total**	140 (20.1)	139 (19.9)	140 (20.1)	139 (19.9)	140 (20.1)	–
**Age, md (r)**		77 (52-92)	78 (52-93)	77 (51-92)	78 (51-94)	77 (52-93)	0.979
**Body Mass Index (BMI), md (r)**		25 (16-39)	26 (16-38)	26 (19-40)	26 (17-39)	25 (16-39)	0.821
**Female**		80 (22.5)	83 (20.6)	74 (20.8)	71 (20.0)	57 (16.1)	0.079
**Race**	**Caucasian**	133 (20.6)	123 (19.1)	140 (20.2)	129 (20.0)	130 (20.2)	0.687
	**Black/African American**	3 (15)	5 (25)	5 (25)	3 (15)	4 (20)	
	**Asian**	4 (14)	10 (34)	5 (17)	6 (21)	4 (14)	
	**Other**	0 (0)	1 (25)	0 (0)	1 (25)	2 (50)	
**Blood-cholesterol lowering medications used**		103 (19.7)	108 (20.6)	108 (20.6)	103 (19.7)	101 (19.3)	0.791
***APOE* ε2 Alleles**	**0**	127 (20.6)	121 (19.6)	126 920.4)	121 (19.6)	122 (19.8)	0.924
	**1**	12 (15.6)	17 (22.1)	13 (16.9)	18 (23.4)	17 (22.1)	
	**2**	1 (25)	1 (25)	1 (25)	0 (0)	1 (25)	
***APOE* ε4 Alleles**	**0**	82 (19.1)	85 (19.8)	86 (20.0)	85 (19.8)	91 (21.2)	0.852
	**1**	48 (21.3)	49 (21.8)	43 (19.1)	45 (20.0)	40 (17.8)	
	**2**	10 (22.7)	5 (11.4)	11 (25.0)	9 (20.4)	9 (20.4)	
**Years of education**	**<12**	0 (0)	2 (13)	7 (47)	3 (20)	3 (20)	**0.034**

	**12**	23 (21.9)	30 (28.6)	14 (13.3)	13 (13.3)	24 (22.9)	
	**12-15**	24 (21.2)	20 (17.7)	29 (25.7)	23 (20.4)	17 (15.0)	
	**≥16**	93 (20.0)	87 (18.7)	90 (19.4)	99 (21.3)	96 (20.6)	
**Smoking**		3 (25)	4 933)	1 98)	3 (25)	1 (8)	0.544
**Moderate-to-heavy alcohol use**		11 (15.1)	12 (16.4)	16 (21.9)	18 (24.7)	16 (21.9)	0.608
**Hypertension**		100 (19.2)	106 (20.4)	114 (21.8)	102 (19.6)	98 (18.8)	0.196
**Atherosclerosis**		5 (11.1)	8 (17.8)	10 (22.2)	14 (31.1)	8 (17.8)	0.258
**Cerebrovascular disease**		29 (20.9)	29 (20.9)	32 (23.0)	20 (14.4)	29 (20.9)	0.461
**Diabetes**		32 (17.8)	47 (26.1)	36 (20.0)	36 (20.0)	29 (16.1)	0.126
**Ischemic heart disease or myocardial infarction**		36 (16.1)	52 (23.3)	52 (23.3)	40 (17.9)	43 (19.3)	0.142
**Malignant neoplasm**		45 (16.1)	62 (22.1)	68 (24.3)	45 (16.1)	60 (21.4)	**0.012**
**Total Cholesterol Trajectory Group**	**1**	50 (15.7)	68 (21.3)	65 (20.4)	70 (21.9)	66 (20.7)	**0.048**
	**2**	78 (25.6)	58 (19.0)	54 (17.7)	52 (17.0)	63 (20.7)	
	**3**	12 (16.2)	13 (17.6)	21 (28.4)	17 (23.0)	11 (14.9)	
**HDL-C Trajectory Group**	**1**	53 (17.5)	67 (22.1)	55 (18.2)	58 (19.1)	70 (23.1)	0.191
	**2**	60 (20.8)	47 (16.3)	65 (22.6)	63 (21.9)	53 (18.4)	
	**3**	27 (25.2)	25 (23.4)	20 (18.7)	18 (16.8)	17 (15.9)	
**non-HDL-C Trajectory Group**	**1**	51 (16.2)	77 (24.4)	61 (19.4)	64 (20.3)	62 (19.7)	0.093
	**2**	81 (23.6)	53 (15.4)	71 (20.7)	65 (19.0)	73 (21.3)	
	**3**	8 (20.0)	9 (22.5)	8 (20.0)	10 (25.0)	5 (12.5)	
**LDL-C Trajectory Group**	**1**	40 (14.0)	68 (23.8)	59 (20.6)	60 (21.0)	59 (20.6)	0.108
	**2**	82 (24.1)	59 (17.4)	66 (19.4)	66 (19.4)	67 (19.7)	
	**3**	18 (25.0)	12 (16.7)	15 (20.8)	13 (18.1)	14 (19.4)	

**ln(Triglycerides) Trajectory Group**	**1**	62 (24.0)	55 (21.3)	43 916.7)	45 (17.4)	53 (20.5)	0.445
	**2**	54 (16.9)	61 (19.1)	71 (22.3)	68 (21.3)	65 (20.4)	
	**3**	24 (19.8)	23 (19.0)	26 (21.5)	26 (21.5)	22 (18.2)	
**MCI Case/Control Cohort (116 cases, 435 controls)**							
**Characteristics**[a]		**VIM Quintile**[b]					***p***[c]
		**1**	**2**	**3**	**4**	**5**	
**Number of cases/controls**	**controls**	85 (19.5)	92 (21.2)	89 (20.5)	87 (20.0)	82 (18.9)	0.460
	**cases**	25 (21.6)	18 (15.5)	21 (18.1)	23 (19.8)	29 (25.0)	
	**total**	110 (20.0)	110 (20.0)	110 (20.0)	110 (20.0)	111 (20.2)	–
**Age, md (r)**		76 (52-92)	75 (51-98)	77 (53-91)	76 (51-94)	76 (53-93)	0.704
**BMI, md (r)**		27 (17-46)	27 (18-43)	27 (17-52)	26 (17-44)	26 (16-41)	0.873
**Female**		58 (22.3)	53 (20.4)	48 (19.5)	54 (20.8)	47 (18.1)	0.531
**Race**	**Caucasian**	104 (20.6)	101 (20.0)	100 (19.8)	99 (19.6)	100 (19.8)	0.552
	**Black/African American**	1 (8)	1 (8)	3 (25)	3 (25)	4 (33)	
	**Asian**	2 (6.9)	7 (24.1)	6 (20.7)	8 (27.6)	6 (20.7)	
	**Other**	3 (50)	1 (17)	1 (16)	0 (0)	1 (17)	
**Blood-cholesterol lowering medications used**		84 (19.2)	87 (19.9)	89 (20.4)	87 (19.9)	90 (20.6)	0.914
***APOE* ε2 Alleles**	**0**	101 (21.0)	94 (19.5)	93 (19.3)	96 (19.7)	97 (20.2)	0.535
	**1**	8 (1.8)	16 (23.5)	17 (25.0)	14 (20.6)	13 (19.1)	
	**2**	1 (50)	0 (0)	0 (0)	0 (0)	1 (50)	
***APOE* ε4 Alleles**	**0**	75 (19.3)	75 (19.3)	83 (21.3)	78 (20.0)	78 (20.0)	0.917
	**1**	31 (21.1)	32 (21.8)	26 (17.7)	29 (19.7)	29 (19.7)	
	**2**	4 (27)	3 (20)	1 (7)	3 (20)	4 (27)	

**Years of education**	**<12**	4 (33)	0 (0)	4 (33)	1 (8)	3 (25)	0.210
	**12**	15 (19.2)	15 (19.2)	12 (15.4)	15 (19.2)	21 (26.9)	
	**12-15**	28 (36.7)	18 (18.1)	25 (23.8)	16 (15.2)	18 (17.1)	
	**≥16**	63 (17.7)	77 (21.6)	69 (19.4)	78 (21.9)	69 (19.4)	
**Smoking**		2 (17)	2 (17)	4 (33)	2 (17)	2 (17)	0.849
**Moderate-to-heavy alcohol use**		9 (14.3)	13 (20.6)	12 (19.0)	13 (20.6)	16 (25.4)	0.702
**Hypertension**		78 (18.3)	85 (19.9)	95 (22.2)	86 (20.1)	83 (19.4)	0.085
**Atherosclerosis**		6 (16.2)	9 (24.3)	3 (8.1)	12 (32.4)	7 (18.9)	0.161
**Cerebrovascular disease**		17 (19.1)	23 (24.5)	13 (13.8)	22 (23.4)	19 (20.2)	0.385
**Diabetes**		35 (21.6)	36 (22.2)	30 (18.5)	37 (22.8)	24 (14.8)	0.256
**Ischemic heart disease or myocardial infarction**		39 (19.2)	43 (21.2)	34 (16.8)	47 (23.2)	40 (19.7)	0.454
**Malignant neoplasm**		40 (17.2)	53 (22.8)	51 (21.9)	53 (22.8)	36 (15.4)	**0.043**
**Total Cholesterol Trajectory Group**	**1**	27 (17.5)	36 923.4)	29 (18.8)	32 (20.8)	30 (19.5)	0.389
	**2**	62 (22.0)	54 (19.2)	63 (22.3)	53 (18.8)	50 (17.7)	
	**3**	21 (18.3)	20 (17.4)	18 (15.6)	24 (21.7)	31 (27.0)	
**HDL-C Trajectory Group**	**1**	62 (20.3)	60 (19.6)	62 (20.3)	62 (20.3)	60 (19.6)	0.988
	**2**	36 (18.8)	38 (19.8)	39 (20.3)	40 (20.8)	39 (20.3)	
	**3**	12 (22.6)	12 (22.6)	9 (17.0)	8 (15.1)	12 (22.6)	
**non-HDL-C Trajectory Group**	**1**	32 (16.9)	50 (26.5)	34 (18.0)	42 (22.2)	31 (16.4)	**0.014**
	**2**	66 (23.2)	47 (16.6)	65 (22.9)	48 (16.9)	58 (20.4)	
	**3**	12 (15.4)	11 (16.7)	11 (14.1)	20 (25.6)	22 (28.2)	
**LDL-C Trajectory Group**	**1**	42 (16.9)	64 (25.7)	50 (20.1)	53 (21.3)	40 (16.1)	**0.036**
	**2**	57 (22.4)	40 (15.7)	54 (21.2)	46 (18.0)	58 (22.8)	

	**3**	1 (23.4)	6 (12.8)	6 (12.8)	11 (23.4)	13 (27.7)	
**ln(Triglycerides) Trajectory Group**	**1**	40 (22.5)	39 (21.9)	30 (16.8)	23 (19.1)	35 (19.7)	0.897
	**2**	50 (17.9)	55 (19.7)	59 (21.1)	57 (20.4)	58 (20.8)	
	**3**	20 (21.3)	16 (17.0)	21 (22.3)	19 (20.2)	18 (19.2)	

[a]Age, BMI and social and medical history information are reported for age at first symptom onset, in cases, or matching age, in controls.

[b]VIM quintile and ln(triglycerides) trajectory group were determined as described in the text using triglyceride levels obtained over the 11-year period prior to and including the year of onset, in cases, or matching age, in controls. Number (percent of row total); for age and body mass index (BMI), median (md) [range(r)].

[c]Kruskal-Wallis test for age and BMI, otherwise χ^2 test; values <0.05 are in bold. An asterisk identifies *p* values remaining significant after Bonferroni adjustment.

Figure S1. Age at first cognitive symptom. (A) AD cases (B) MCI cases. Compare to **Table 1.**

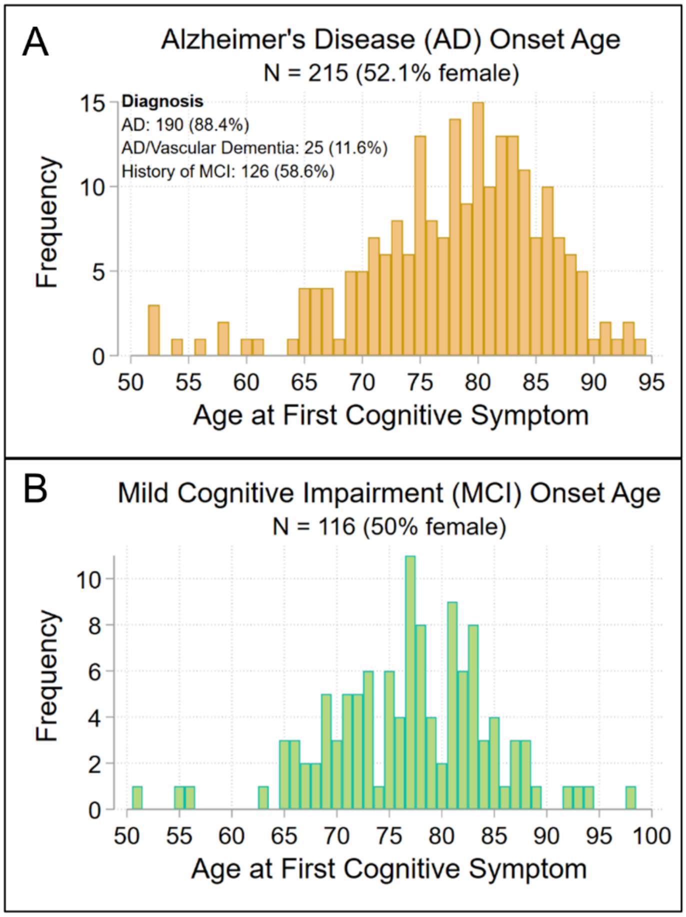

Figure S2. **Lipid measurements in the AD and MCI case-control cohorts.**

Group-based trajectory models were developed using lipid measurements obtained a decade prior to the year of onset (cases) or year of age-match (controls). Panels **A** (AD) and **C** (MCI) show the percentages of cases and controls who had lipid measurements each year. Study inclusion criteria required subjects to have at least three lipid measurements. Panels **B** (AD) and **D** (MCI) show how the number of lipid measurements was distributed in cases and controls.

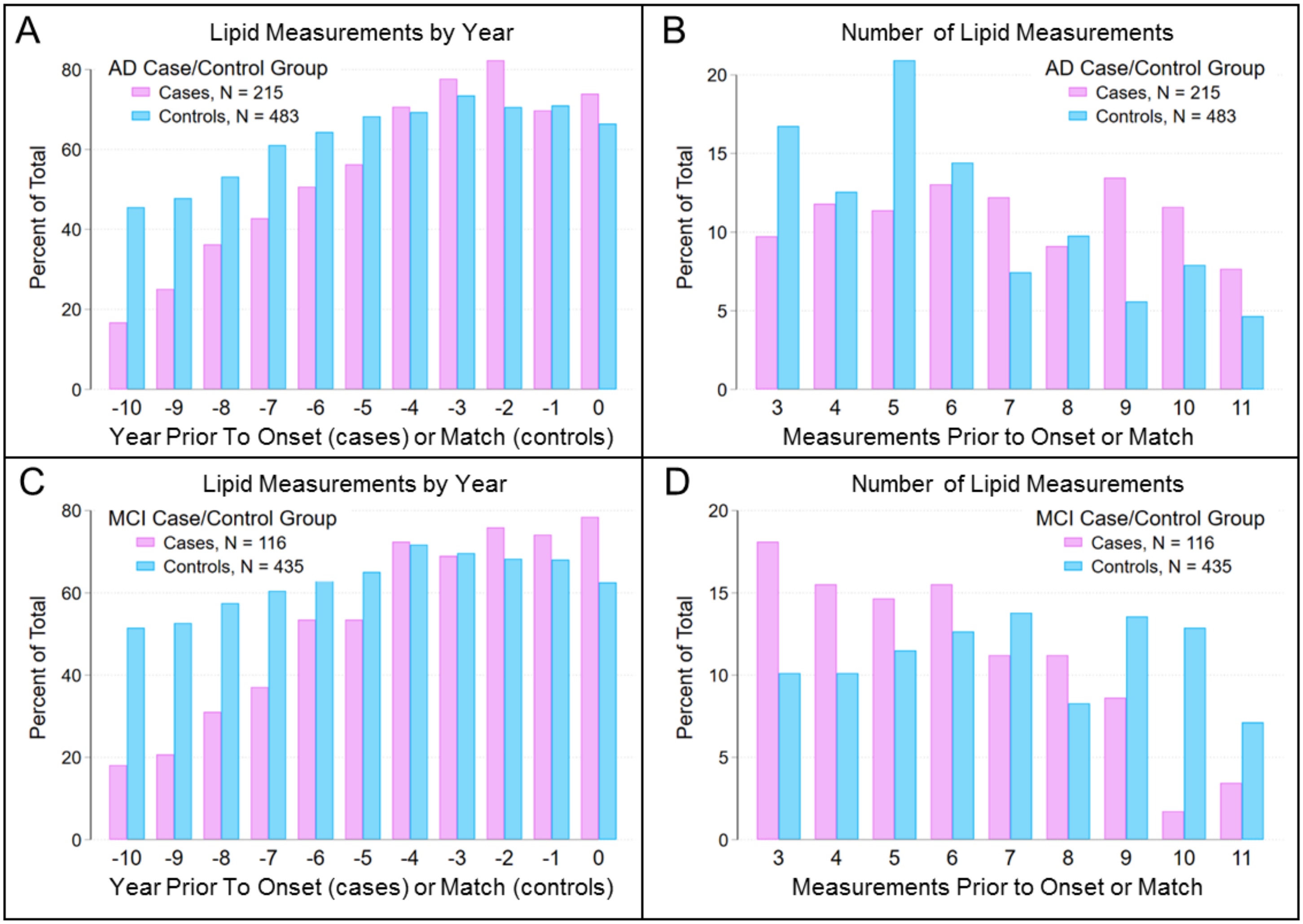

Figure S3. Contributions of HDL-C trajectory groups and the lowest quintile of total non-HDL-C VIM to MCI risk, relative to an AD polygenic risk score. The covariate-adjusted MCI-risk model including polygenic risk scores summarized in **Figure 4B** and **Table 3** was used to estimate contributions to MCI risk relative to an AD polygenic risk score (PRS, PGS004092). (**A**) Predictive margins for 5th, 50th, and 95th percentile AD PRS scores for subjects aged 65-85, at mean values of other covariates. (**B**) Predictive margins of HDL-C trajectory groups at age 75 over a range of AD PRS scores and at mean values of other covariates. (**C**) Predictive margins of the lowest quintile of total-cholesterol VIM at age 75 over a range of values of AD PRS scores and at mean values of other covariates. Lines illustrate predicted MCI risk; shaded areas indicate 95% confidence intervals. In panel **D,** kernel-density plots obtained using an Epanechnikov kernel function illustrate the distribution of the AD PRS scores in MCI cases and matched controls. A Kolmogorov-Smirnov test evaluated the equality of the distribution of the PRS in cases and controls.

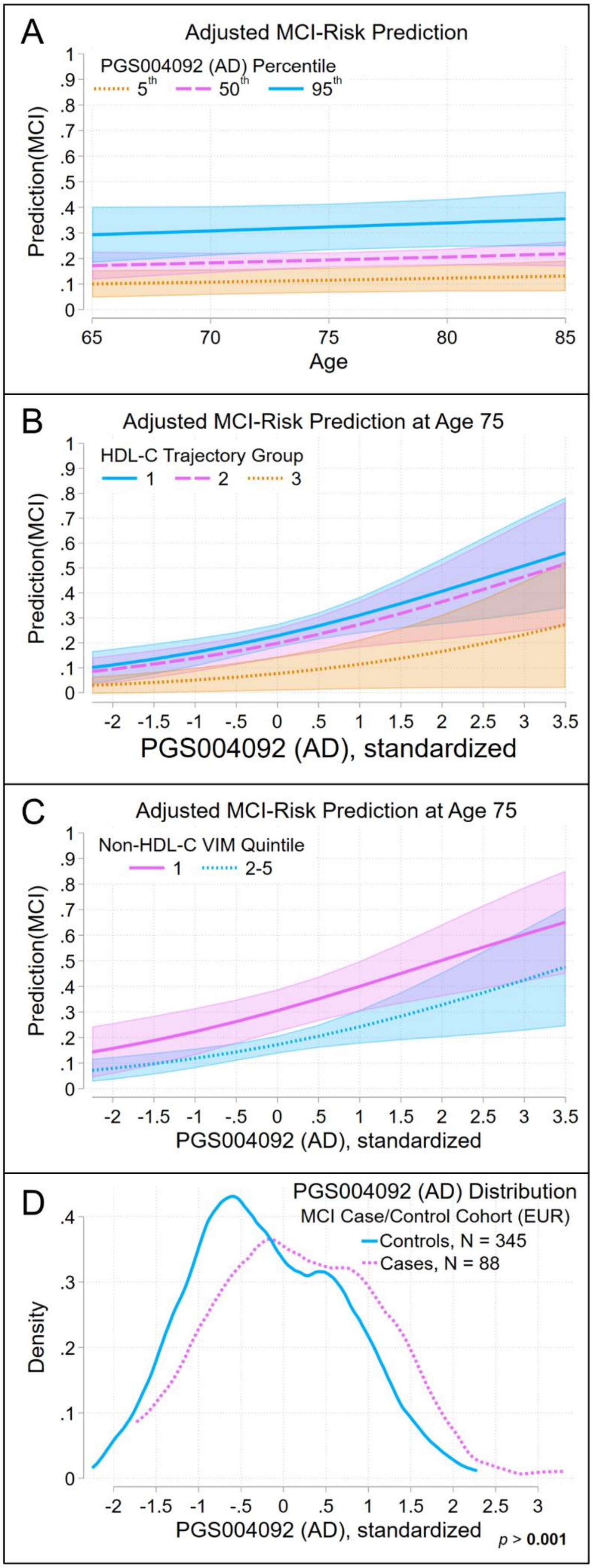

Figure S4. Contributions of HDL-C trajectory groups and the lowest quintile of non-HDL-C VIM to MCI risk, relative to *APOE*-ε4 genotype. The covariate adjusted MCI-risk model including *APOE* genotypes summarized in **Figure 4F** and **Table 3** was used to estimate contributions to MCI risk, relative to *APOE*ε4 genotype. Panels at left show the predictive margins for HDL-C trajectory groups for subjects aged 50-85 and at mean values of other covariates in (**A**) non-*APOE*-ε4 genotypes, (**C**) *APOE*-ε4 heterozygotes, and (**E**) *APOE*-ε4 homozygotes. Panels at right show the predictive margins for the lowest quintile of non-HDL-C VIM for ages 50-85 and at mean values of other covariates in (**B**) non-*APOE*-ε4 genotypes, (**D**) *APOE*-ε4 heterozygotes, and (**F**) *APOE*-ε4 homozygotes. Lines illustrate the predicted MCI risk; shaded areas indicate 95% confidence intervals. Bar plots (bottom panels) show the distribution of (**G**) HDL-C trajectory groups and (**H**) lowest total-cholesterol VIM quintile across *APOE*-ε4 genotypes. χ^2-contingency tests evaluated whether the distribution of HDL-C trajectory groups or non-HDL-C VIM quintile groups differed across *APOE*-ε4 genotypes.

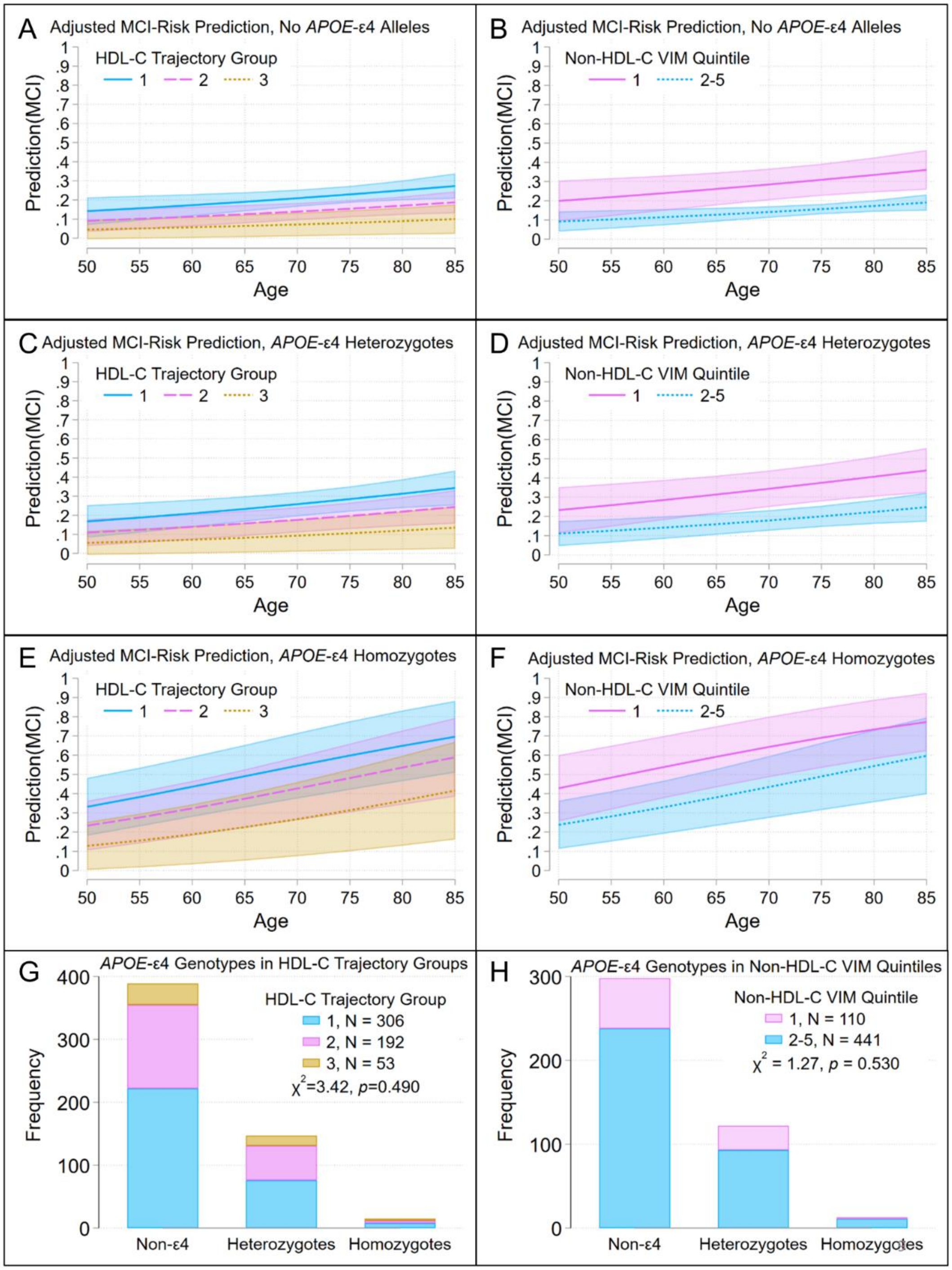

Supplement: Supplemental Material [file mmc1.pdf]
